# Genome sequencing of adapted diploid potato clones

**DOI:** 10.3389/fpls.2022.954933

**Published:** 2022-08-08

**Authors:** Sai Reddy Achakkagari, Maria Kyriakidou, Kyle M. Gardner, David De Koeyer, Hielke De Jong, Martina V. Strömvik, Helen H. Tai

**Affiliations:** ^1^Department of Plant Science, McGill University, Sainte-Anne-de-Bellevue, QC, Canada; ^2^Fredericton Research and Development Centre, Agriculture and Agri-Food Canada, Fredericton, NB, Canada

**Keywords:** diploid potato, breeding, genome sequencing, deleterious alleles, *StCDF1*, potato

## Abstract

Cultivated potato is a vegetatively propagated crop, and most varieties are autotetraploid with high levels of heterozygosity. Reducing the ploidy and breeding potato at the diploid level can increase efficiency for genetic improvement including greater ease of introgression of diploid wild relatives and more efficient use of genomics and markers in selection. More recently, selfing of diploids for generation of inbred lines for F1 hybrid breeding has had a lot of attention in potato. The current study provides genomics resources for nine legacy non-inbred adapted diploid potato clones developed at Agriculture and Agri-Food Canada. *De novo* genome sequence assembly using 10× Genomics and Illumina sequencing technologies show the genome sizes ranged from 712 to 948 Mbp. Structural variation was identified by comparison to two references, the potato DMv6.1 genome and the phased RHv3 genome, and a k-mer based analysis of sequence reads showed the genome heterozygosity range of 1 to 9.04% between clones. A genome-wide approach was taken to scan 5 Mb bins to visualize patterns of heterozygous deleterious alleles. These were found dispersed throughout the genome including regions overlapping segregation distortions. Novel variants of the *StCDF1* gene conferring earliness of tuberization were found among these clones, which all produce tubers under long days. The genomes will be useful tools for genome design for potato breeding.

## Introduction

Achieving global food security sustainably under climate change is a key challenge facing society. Potato, *Solanum tuberosum* L., is currently the third most important crop for human consumption (International Potato Center, 2021). It has seen production growth in recent years, particularly in developing regions of the world, and is considered to be a sustainable global food security crop for climate smart agriculture (CSA) (Devaux et al., 2014). Improving potato through breeding is part of a strategy to increase resiliency of the crop to stress including drought, heat, pests, pathogens and extreme weather events (Dahal et al., 2019). Recently, there has been a resurgence of interest in introgression breeding to address trait improvement for climate change adaptation, sustainability and overcoming yield plateaus (Hao et al., 2020). Development of improved potato varieties can be enhanced through introgression of genes from over 100 wild *Solanum* relatives that extend from the United States to Chile (Spooner et al., 2014).

Cultivated *S. tuberosum* is mostly tetraploid (2n = 4x = 48) whereas most of the intercrossing wild *Solanum* species are diploid (2n = 2x = 24) (Spooner et al., 2014; Bethke et al., 2019). Diploid potato breeding schemes enable introgression of diploid wild relatives as well as more efficient utilization of genomic and marker selection strategies to increase beneficial allele combinations. Ploidy manipulation methods were established years ago to introgress beneficial genes from diploids to tetraploids (Peloquin et al., 1991). Crossing tetraploids with haploid inducers to generate 2× dihaploids enables intercrossing with wild diploid species. After multiple cycles of selection for adaptation and required agronomic traits, superior diploid hybrids can be crossed with meiotic mutants producing 2n gametes to generate tetraploid progeny. Alternatively, the chromosome number of diploid clones can be doubled using application of colchicine to plant material propagated *in vitro* to reconstitute tetraploids. More recently, diploid clones carrying mutations in the *Sli* self-incompatibility locus have been used to generate inbred lines with a goal to develop diploid F_1_ hybrid breeding for potato (Lindhout et al., 2011; Jansky et al., 2016). A hurdle is the high level of inbreeding depression in diploid potato (De Jong and Rowe, 1971; Zhang et al., 2019).

As an outcrossing autotetraploid with low levels of recombination (Bradshaw, 2021), the potato genome accumulates high levels of deleterious alleles, which contribute to inbreeding depression (Charlesworth and Willis, 2009; Zhang et al., 2019). Inbreeding increases homozygosity leading to exposure of recessive deleterious alleles to selection and purging. However, overdominance and pseudo-overdominance can lead to blocks of loci carrying heterozygous deleterious alleles fixed in repulsion which counteracts purging (Waller, 2021). Increase in linkage disequilibrium also increases with inbreeding, which also reduces purging of deleterious alleles. To efficiently select diploid germplasm for inbreeding with reduced deleterious alleles, genomic regions affected by these deleterious alleles need to be identified. Genome scanning for deleterious alleles and segregation distortions was previously reported by others for potato (Zhang et al., 2019, 2021; Zhou et al., 2020; Hoopes et al., 2022). The information was used to develop genome design strategies for inbreeding potato including precision targeting, leading to faster development of inbred lines for hybrid breeding of F_1_ progeny that demonstrated heterosis (Zhang et al., 2021).

Legacy adapted diploid breeding clones are carried by several potato breeding programs around the world. The backgrounds of these clones include a history of dihaploid extraction, introgression with wild relatives and partial inbreeding, and they serve as resources for diploid F_1_ hybrid breeding. Here, we present the genome sequences of nine diploid clones from the legacy diploid breeding collection of Agriculture and Agri-Food Canada, and utilize this information to advance genome design for potato breeding.

## Materials and methods

### *De novo* genome assembly

A panel of nine diploid clones (07506-01, 12120-03, DW84-1457, 12625-02, 08675-21, H412-1, W5281.2, 11379-03, and 10908-06) from the breeding collection of Agriculture and Agri-Food Canada were selected for this study. The pedigrees for these clones is found in Supplementary Figure 1. Seven of the clones are also publicly available through the United States NRSP-6 Potato Genbank in Sturgeon Bay, WI, United States [Genbank id numbers are in brackets, 07506-01 (BS 280), DW84-1457 (BS 288), 08675-21 (BS 281), H412-1 (BS 279), W5281.2 (GS 217), 11379-03 (BS 287), and 10908-06 (BS 285)]. Information about the plant material, DNA extraction and sequencing using 10× Genomics’ GemCode technology^1^ can be found in Achakkagari et al. (2021a). The 10× Genomics linked reads were used for *de novo* assembly of each genome sequence using Supernova™ genome assembler (Weisenfeld et al., 2017). The pseudohap2 option was used to generate both haplotypes and the first pseudo haplotype was used in all subsequent analyses. The organelle sequences and contaminants were removed from the assemblies by aligning to potato plastomes and mitogenomes as well as to the Univec database^2^ (Kitts et al., 2011) using BLAST + v2.7.1 (Camacho et al., 2009). The filtered contigs were corrected for structural errors using Tigmint v1.1.2 (Jackman et al., 2018) and assembled into scaffolds using ARCS v1.1.1 (Yeo et al., 2018). To evaluate the genome assemblies, BUSCO v5.2.2 (Simão et al., 2015) was used to detect the gene content and QUAST v5.0.2 (Gurevich et al., 2013) was used for the assembly statistics.

#### Detection of structural variants

Structural variants such as CNVs and SNPs present in each genome were detected against the references DMv6.1 genome from *S. phureja* DM1-3 516 R44 (Potato Genome Sequencing Consortium, 2011; Pham et al., 2020) and RHv3 genome from *S. tuberosum* RH89-039-16 (Zhou et al., 2020). The CNVs were determined using Longranger WGS (Marks et al., 2019) with freebayes as the haplotype caller (Garrison and Marth, 2012). The SNPs were detected by mapping the reads to the two reference genomes individually. First, the raw reads were filtered and error-corrected using Longranger basic. The filtered reads were mapped to the DMv6.1 and RHv3 using BWA-MEM v0.7.17 (Li, 2013), and only proper read pairs were kept using Samtools v1.13 (Li et al., 2009). The duplicates were marked with PicardTools v.2.23.3 (Picard Toolkit, 2019). The variants were called using freebayes v1.2.0 (Garrison and Marth, 2012), and filtered using vcffilter from the vcflib package (Garrison et al., 2021). A minimum mapping quality of 30 and minimum base quality of 30 was used for freebayes. Also, the following filters were applied to remove low quality sites (QUAL < 20), and sites with depth (DP < 4 and > 50). An allele balance number of ≥ 0.3 and ≤ 0.7 is considered for a heterozygous allele, and ≥ 0.9 and ≤ 0.1 for a homozygous allele (Zhang et al., 2019). The SNPs were annotated using BEDTools v2.29.2 (Quinlan and Hall, 2010). A phylogenetic tree was constructed from the SNP data against the DMv6.1 to see the relationship between the clones. For its construction, the individual bam alignments of each clone, including RHv3, were merged together and SNPs were called using freebayes. The filtered variants were converted to a nexus format using vcf2phylip v2.8 (Ortiz, 2019) requiring a minimum of four samples at a locus. A parsimonious phylogenetic tree was constructed using paup v4.0a (Swofford, 2008) with 1000 bootstrap replicates. The tree was visualized with FigTree v1.4.4.^3^

### Heterozygosity and deleterious allele analysis

Trimmed 10× Genomics sequencing reads were used for the calculation of the percentage of heterozygosity in the genomes. For this, jellyfish v2.2.10 (Marçais and Kingsford, 2011) was first used to compute the histogram of the k-mer frequencies. The final k-mer count histogram per genome was used within the GenomeScope 2.0 online platform (Ranallo-Benavidez et al., 2020). For deleterious allele analysis, a genomic database with SIFT predictions for DMv6.1 and RHv3 were created separately using the SIFT4g algorithm v2.0.0 (Vaser et al., 2016). Two protein databases were generated for the RHv3 for each of the haplotypes. The deleterious mutations in each genome against DMv6.1 and RHv3 were predicted using the SIFT4g annotator (Vaser et al., 2016). The mutations with a SIFT score of ≤ 0.05 are predicted to be deleterious, and mutations tolerated if the SIFT score is >0.05. Low-confidence deleterious calls were filtered out from the output. The reference genomes were divided into 5 Mb bins using BEDTools v2.30.0 (Quinlan and Hall, 2010) and for each bin, the percentage (%) of genes with deleterious alleles was calculated as follows:% of genes with deleterious alleles = (number of genes affected by deleterious alleles/total no. of genes) × 100. Heterozygosity of deleterious alleles was determined using the VCF files from the SNP analysis and % genes with heterozygous deleterious alleles = (number of heterozygous deleterious alleles/total no. of genes) × 100.

### Segregation distortion analysis

Ninety progeny from a cross between 12120-03 (female parent) and 07506-01 (male parent) were genotyped using the SolCap 8303 Infinium chip (Tai et al., 2018). The SNP genotyping data is available at doi: 10.5061/dryad.2547d7wt8. Segregation distortion was determined for SNP markers that were heterozygous in each parent using a chi-square test to assess deviation from the expected 1:2:1 (homozygous:heterozygous:homozygous) Mendelian genotypic class frequencies. Ratios for each SNP marker analyzed using chisq.test in R v4.1.1 (R Development Core Team, 2021). To account for multiple testing, the Bonferroni correction method was applied to the segregation data using p.adjust in R v4.1.1 (Dai et al., 2017; R Development Core Team, 2021). The −log_10_ of the Bonferroni adjusted *p*-value from the chi-square test was determined for each marker and averaged over 1 Mb bins. A −Log_10_ (*p*-value) >1.2 was considered to have segregation distortion.

#### Kompetitive allele specific PCR™ genotyping

Genotyping of the *Sli* locus on chromosome 12 using Kompetitive Allele Specific PCR (KASP)™ markers was conducted with genomic DNA from the nine diploid clones using the methods described by Clot et al. (2020). KASP markers ST4_03ch12 58962561 and ST4_03ch12 59040898 were used and assays were run according to Kaiser et al. (2021) on a Light Cycler 480 Real-Time PCR system (Roche, Germany). The 3.25 μl reaction system with 1.5 μl v4.0 2× Low ROX KASP Mastermix (LGC Genomics, Beverly, MA, United States), 0.05 μl KASP Assay by Design primer and 1.7 μl of 15–10 ng/μl genomic DNA was used. PCR conditions were 95°C 10 min, followed by 10 cycles of touch down PCR from 65°C to 57°C with 0.8°C decrease per cycle, followed by 37 cycles of 95°C for 20 s and 58°C for 1 min. Genotyping of the *StCDF1* locus on chromosome 5 was done using Kompetitive Allele Specific PCR KASP markers using a commercial service provider, Intertek ScanBi Diagnostics AB (Alnarp, Sweden).^4^ The marker sequences are in Supplementary Table 6. Predicted DNA secondary structure of KASP PCR product amplified by the common primer and the snpST00091 primer were determined using the Vector Builder webtool.^5^

## Results

### Agriculture and Agri-Food Canada adapted diploid clones

Nine diploid potato clones from the breeding collection of Agriculture and Agri-Food Canada were selected for a range of traits including maturity, tuber quality and appearance, pigmentation, fertility, and resistance to disease (Table 1 and Supplementary Figure 1). The clones were genotyped for the *Sli* mutant allele conferring self-compatibility using the KASP™ markers described by Clot et al. (2020), ST4_03ch12 58962561 and ST4_03ch12 59040898. Both of the markers showed that H412-1 was heterozygous for the *Sli* mutant allele (Table 2). None of the other clones were found to carry *Sli*.

**TABLE 1:** Description of the nine diploid potato clones selected for this study.

Clone #	NRSP-6	Parents	Traits
W5281.2	GS 217	phu 195198 × W1 dihaploid from Katahdin	*S. phureja* × *S. tuberosum* hybrid. Relatively low yield. Purple skin, deep eyes, excellent chip color. Moderate scab resistance. Poor male fertility.
H412-1	BS 279	06026-08 x (bulk 2× tbr)	*S. tuberosum*. Moderate yield and size. Mid to late vine maturity. Mediocre chipper, scab susceptible. Heterozygous for the *Sli* allele conferring self-compatibility.
07506-01	BS 280	W9306.2 × (bulk 2× hybrid)	*S. tuberosum* with pedigree including *S. phureja*. Excellent (oblong) tuber type and size. Mediocre chipper. Mid to late vine maturity. Susceptible to most diseases. Excellent male fertility.
08675-21	BS 281	06824–02 × 07506–01	*S. tuberosum* with pedigree including *S. phureja*. Excellent tuber type, size, yield, chip color and scab resistance. Mid to late vine maturity. Good flowering and male fertility.
11379-03	BS 287	BPH32-05 × 09941-05	*S. tuberosum* with pedigree including *S. stenotomum* or *S. phureja*. Excellent tuber size and (long) type. Mid to late vine maturity. Fairly good chipper. Lots of flowers, good male fertility.
10908-06	BS 285	09507-03 × H412-1	*S. tuberosum* with a pedigree including wild Argentine diploids. Mid to late vine maturity. Good chip color. Resistance to PVY. H412-1 is the parent indicated in the breeding records at AAFC, but this is not supported by SNP data (Figure 3).
DW84-1457	BS 288	DW81-1470 × DW80-2031	*S. tuberosum* with pedigree including the following wild species *S. acroglossum*, *S. demissum*, *S. gourlayi*, and *S. stoloniferum*. PLRV, PVX and PVM resistance. Good table and processing qualities. Poor male fertility.
12625-02		2x (V-2)7 (Ry adg) × 11379-03	*S. andigena* with pedigree including *S. tuberosum* and *S. stenotomum* or *S. phureja*. PVY-immune. Good male fertility.
12120-03		09113-11 × 09753-01	*S. tuberosum* with pedigree including *S. phureja* and *S. stenotomum*. Round tubers and pink skin pigmentation. Scab and *Verticillium dahliae* resistance.

**TABLE 2:** *Sli* KASPr genotyping with ST4_03ch12 58962561 (Sli 561) and ST4_03ch12 59040898 (Sli 898).

Clone_ID	Sli 561	Sli 898
07506-01	A:A	A:A
12120-03	A:A	A:A
10908-06	A:A	A:A
11379-03	A:A	A:A
08675-21	A:A	A:A
**H412-1**	A:B	A:B
W5281.2	A:A	A:A
DW84-1457	A:A	A:A

The B allele is the Sli self-incompatibility mutant.

### *De novo* genome assembly of adapted diploid clones

The genome sequences of the panel of nine diploid potato clones were *de novo* assembled using sequencing data from 10× Genomics, which resulted in assemblies ranging from 712 to 948 Mbp in size (Supplementary Table 1). The 11379-03 genome has the largest assembly with 948,106,575 bp and 10908-06 with 712,637,036 bp has the smallest assembly. The 08675-21 genome has the less fragmented assembly (contig N50 46,214 bp), followed by H412-1 (contig N50 45,637 bp), whereas the 10908-06 has the most fragmented assembly (contig N50 of 6,726 bp) (Supplementary Table 1). The completeness of the genome assemblies were evaluated by BUSCO (Simão et al., 2015). The results range between 77.6% of the BUSCO core Solanales ortholog genes in 10908-06 up to 92.4% in H412-1 (Supplementary Figure 1). The completeness of the 10908-06 is affected by its highly fragmented assembly. Overall, based on the N50 and the proportion of the completed genes present, 08675-21 and H412-1 have the best genome assemblies (Supplementary Table 1 and Supplementary Figure 2). The plastomes and mitogenomes for these clones were previously reported (Achakkagari et al., 2021a,b).

**FIGURE 1:**
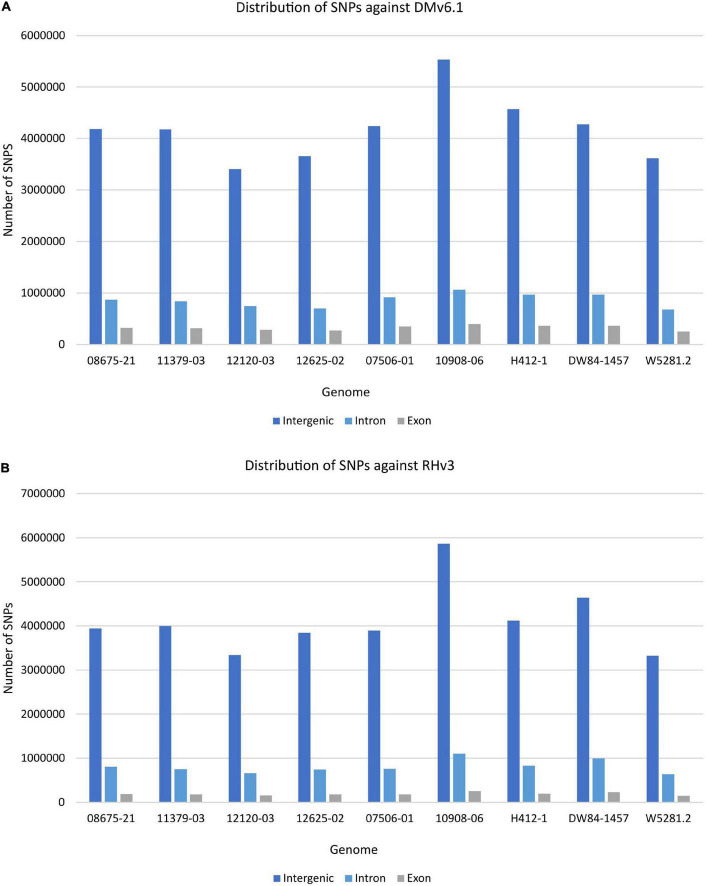
Distribution of SNPs in each genome in the intergenic, intron, and exon regions when compared against **(A)** DMv6.1 and **(B)** RHv3.

**FIGURE 2:**
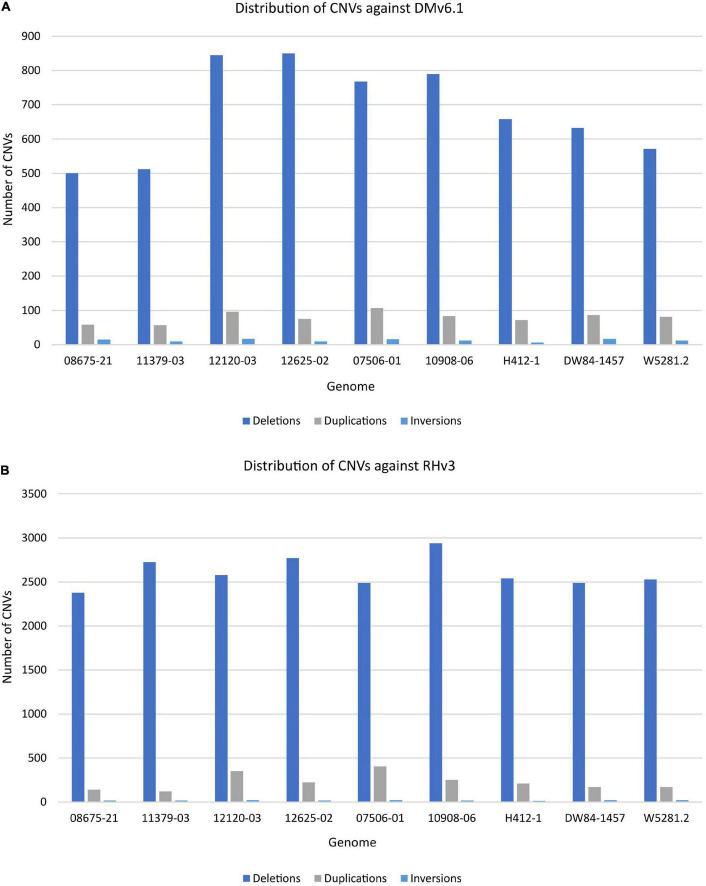
Distribution of CNVs in each genome when compared against **(A)** DMv6.1 and **(B)** RHv3.

The extent of variation within these clones was identified by looking for structural variations against the DMv6.1 and RHv3 reference genomes (Chen et al., 1976; Potato Genome Sequencing Consortium, 2011; Pham et al., 2020; Zhou et al., 2020). The 10908-06 genome harbors the highest number of SNPs when compared against DMv6.1 (Supplementary Table 2). Similar results are observed when compared against RHv3 as well (Supplementary Table 3). The W5281.2 and 12120-03 genomes harbor the lowest numbers of SNPs when compared against DMv6.1 and RHv3. A majority of the SNPs are present in intergenic and intron regions in each genome as shown in Figure 1. Detection of CNVs revealed a greater number of deletions in each genome against both reference genomes compared to duplications and inversions. Overall, the 12120-03 and 12625-02 genomes have the highest number of CNVs against DMv6.1, whereas the 10908-06 and DW84-1457 genomes have the highest number of CNVs against RHv3. The number of CNVs in each genome are shown in Figure 2. The CNVs were compared among the nine clones and only a few deletions were found with exact breakpoints shared by all. The chr7_1:59960000-62700000, chr2_2:46260000-47860000 deletions from RHv3, and scaffold_36:1-70000, scaffold_310:1-60000, scaffold_303:1-100000, scaffold_295:1-70000, scaffold_268:1-60000, scaffold_252:1-60000, scaffold_138:1-60000 deletions from DMv6.1 are present in all the nine clones. Heterozygosity was estimated using jellyfish (Marçais and Kingsford, 2011) and was found to be highest for 12625-02 and lowest for 12120-03 (Supplementary Table 4).

**FIGURE 3:**
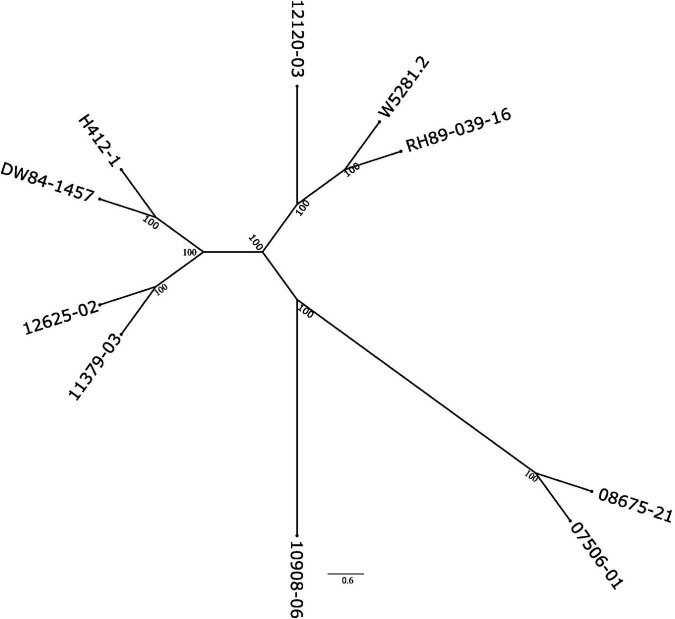
Phylogenetic tree of the nine diploid clones based on the SNP analysis using the DMv6.1 as a reference.

To see how close the clones are to each other, a phylogenetic tree based on SNP data against the DMv6.1 was constructed as shown in Figure 3. Clone 10908-06 shows the greatest distance from the other clones in the tree. The genotyping-by-sequencing (GBS) SNP analysis has similarly shown that 10908-06 is more distant from the other diploids in the study (data not shown). The tree also provides support for the close relationships between 12625-02 and 11379-03, and 08675-21 and 07506-01 that is described in the pedigree information supplied by the breeder (Table 1). However, a close relationship between H412-1 and 10908-06 as documented in the pedigree information from breeding records (Table 1) is not supported by the phylogenetic tree analysis (Figure 3) or GBS analysis (data not shown).

### Analysis of deleterious alleles

**FIGURE 4:**
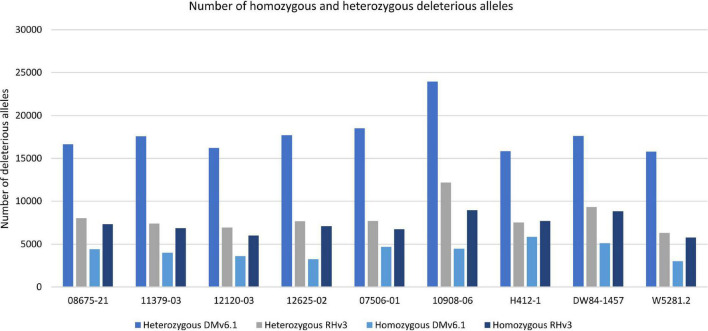
Number of homozygous and heterozygous deleterious alleles in each genome against DMv6.1 and RHv3.

**FIGURE 5:**
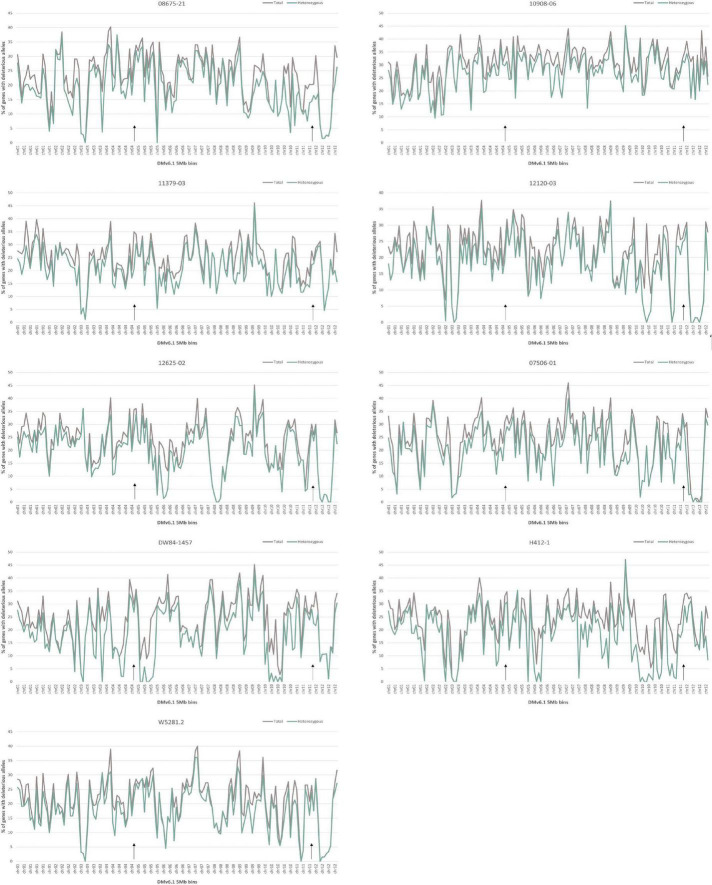
Percentage of deleterious allele affected genes in 5 Mb bins of DMv6.1 in each genome. Black arrows over the *x*-axis indicate locations of the genes *StCDF1* on chr05 and *Sli* on chr12.

Analysis of deleterious alleles based on prediction of the effects of amino acid substitutions was done using SIFT (Vaser et al., 2016). Overall, 10908-06 has the highest number of deleterious alleles and W5281.2 has the lowest when compared against DMv6.1 and RHv3 (Figure 4). Inbreeding can have the advantage of fixing beneficial alleles in homozygosity. However, when beneficial alleles are linked to heterozygous deleterious alleles, inbreeding depression can occur when deleterious alleles also become homozygous. Regions of a genome high in heterozygous deleterious alleles may present challenges for inbreeding. Heterozygosity of deleterious alleles was determined using the SNP data. The genomes were divided into 5 Mb bins using the DMv6.1 genome as a reference and percent total deleterious alleles and percent heterozygous deleterious alleles in each bin was plotted (Figure 5). The percent total deleterious alleles was variable across the genome for each clone and the pattern of deleterious alleles varied between clones. The percent heterozygous deleterious alleles mostly followed the pattern for percent total deleterious alleles. Some regions with high percent total deleterious alleles but low percent heterozygosity were identified where the deleterious alleles are mostly homozygous. The percent heterozygous deleterious alleles was also compared to the gene density across the genome (Supplementary Figure 3). Regions high in deleterious alleles were associated with both gene dense and gene sparse regions. The patterns for deleterious alleles showed differences when the RHv3 haplotype 1 and haplotype 2 protein databases were used for the SIFT analysis instead of DMv6.1 (Supplementary Figures 4, 5). An example is a region on chromosome 12 which had low % of deleterious alleles when the SIFT analysis was done with DMv6.1 and RHv3 haplotype 1 protein databases but not RHv3 haplotype 2. It was also notable that H412-1 which carried the *Sli* mutant conferring self-compatibility was in a region showing high levels of heterozygous deleterious alleles.

**FIGURE 6:**
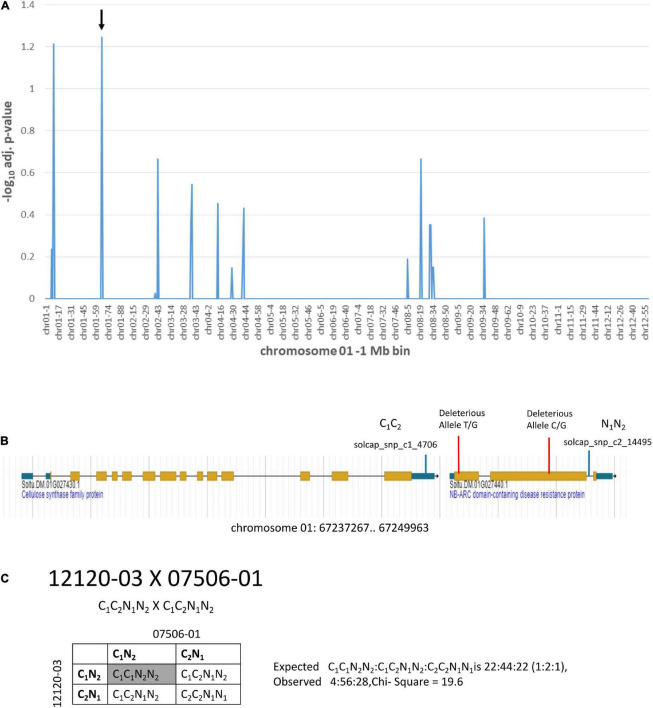
Segregation distortion on chromosome 1 from a cross of 12120–03 × 07506–01. **(A)** Markers heterozygous in both parents were tested for deviation from the expected 1:2:1 segregation ratio of progeny using the chi-square test. The *y*-axis is the –log_10_ Bonferroni adjusted *p*-value from the chi-square test for each of the markers. The *x*-axis shows the genome divided into 1 Mb bins which is labeled with the chromosome location-bin number. The -log10 adjusted *p*-value ≥ 1.2 was the threshold for segregation distortion. The arrow indicates the location of the solcap_snp_c2_14495 marker. This marker is in a gene that has two predicted deleterious alleles. **(B)** The region indicated by the arrow is shown in the DMv6.1 genome browser. **(C)** Progeny genotypes from a cross of 12120–03 × 07506–01. The solcap_snp_c2_14495 and solcap_snp_c1_4706 markers were adjacent to each other and both showed segregation distortion. The progeny genotype in the gray box has significantly reduced frequency compared to expected.

Additional information on deleterious alleles can be gleaned from genetic mapping populations where distorted segregation is observed with loss of individuals carrying loci with homozygous deleterious alleles. Two of the diploid clones, 12120-03 and 07506-01, were parents of a segregating population. The population was used for a genetic mapping study (Tai et al., 2018). Markers present in both parents in heterozygosity will produce progeny with a 1:2:1 segregation ratio of AA:Aa:aa. Loss of homozygous genotypes in progeny are indicative of deleterious alleles that cause lethality and will lead to segregation distortion and a deviation from the 1:2:1 segregation ratio. Chi-square analysis of expected vs. observed segregation ratios for markers heterozygous for both 12120-03 and 07506-01 was done (Figure 6 and Supplementary Table 5). The -log10 adjusted *p*-value ≥ 1.2 was the threshold used for categorization of segregation distortion. There were two locations with markers showing segregation distortion (Figure 6A and Supplementary Table 5). At one location the solcap_snp_c2_14495 marker and the adjacent solcap_snp_c1_4706 marker both showed segregation distortion. The solcap_snp_c2_14495 marker was in the Soltu.DM.01G027440.1 NB-ARC domain-containing disease resistance protein gene which was also found to carry two SNPs predicted to be deleterious mutations (Figure 6B and Supplementary Table 5). The solcap_snp_c1_4706 marker was in the adjacent upstream gene, Soltu.DM.01G027430.1 Cellulose synthase family protein. No deleterious alleles were found in this gene. The two genes were in close proximity and it is hypothesized that the linkage to the deleterious mutations in Soltu.DM.01G027440.1 NB-ARC domain-containing disease resistance protein gene were causing segregation distortion of the adjacent Soltu.DM.01G027430.1 Cellulose synthase family protein gene. Analysis of the segregation ratios of the two genes supports this hypothesis (Figure 6C). The results also suggest that the Soltu.DM.01G027440.1 NB-ARC domain-containing disease resistance protein gene is required for survival as deleterious mutations cause the loss of homozygous progeny.

### *StCDF1* loci

**FIGURE 7:**
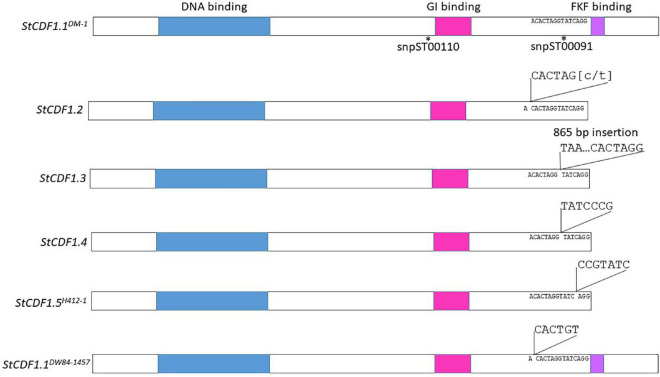
Insertion mutations of *StCDF1* alleles. The location of KASP™ markers snpST00110 and snpST00091 are shown. The DNA binding domain is indicated in blue, GI binding domain is pink and FKF binding domain is purple.

Contigs from the genome sequence of the diploid clones were aligned to *StCDF1.1* from DMv6.1 and it was found that DW84-1457, 11370-03 and 10908-06 encoded new variant alleles (Supplementary Figure 6). These variant alleles were predicted to produce full-length proteins without the truncations present in previously described alleles, *StCDF1.2*, *StCDF1.3*, and *StCDF1.4* (Kloosterman et al., 2013; Gutaker et al., 2019). For this reason, the *StCDF1.1* alleles were named using nomenclature with the clone name in superscript as follows: *StCDF1.1*^DM–1^, *StCDF1.1*^DW84–1457^, *StCDF1.1*^11379–03^, and *StCDF1.1*^10908–06^. One of the variant alleles, *StCDF1.1*^DW84–1457^, carried a six-nucleotide insertion in a similar region as the insertions of the other variant alleles (Figure 7 and Supplementary Figures 6, 7). The *StCDF1.1*^DW84–1457^ variant encodes all functional domains of the protein but has additional leucine and serine residues upstream of the FKF domain (Figure 7). *StCDF1.1*^11379–03^ and *StCDF1.1*^10908–06^ carried alleles missing a serine at amino acid 322, which did not affect the rest of the protein sequence. H412-1 carried a novel *StCDF1* allele, which was named *StCDF1.5*^H412–1^, that had an insertion that truncated the FKF domain, similarly to *StCDF1.2*, *StCDF1.3*, and *StCDF1.4* (Figure 7 and Supplementary Figure 6). The rest of the sequence variations found among the diploid clones resulted in single amino acid substitutions. The snpST00091 KASP marker that detects the *StCDF1.1*^DM–1^ or *StCDF1.4* alleles were used for genotyping the diploid clones (Supplementary Tables 6, 7). The marker was not detected in DW84-1457, which concurs with disruption of primer binding in the KASP genotyping assay due to the six nucleotide insertion found in *StCDF1.1*^DW84–1457^. The results also indicate that the *StCDF1.1*^DW84–1457^ allele is homozygous in DW84-1457. All other diploids showed presence of snpST00091 marker in homozygosity or heterozygosity. The sequence reads from the clones were aligned to the *StCDF1.3* allele, which has an 865 bp insertion. The 11379-03, DW84-1457, and H412-1 genomes did not have reads aligning to the 865 bp insertion suggesting an absence of the *StCDF1.3* allele. The snpST00091 and snpST00092 KASP genotyping assays also show that these clones do not carry *StCDF1.3* (Supplementary Table 7). Also, a large genomic region containing the *StCDF1* gene was found to carry deletions in all the nine clones when contigs were mapped against the DMv6.1. Deletions were also found when mapping contigs against RHv3 but not always against the same haplotype. These results suggest high levels of sequence variation in the genome around *StCDF1*. Greater sequencing depth in future studies will be required to map these deletions to functional regions with accuracy.

## Discussion

Realizing advantages of diploid inbreeding for potato will require development of diploid clones that capture genetic diversity required for different market classes, in addition to resistance to abiotic and biotic stress (Jansky et al., 2016). Reduction of tetraploid breeding clones to diploids is a strategy to generate diploid germplasm, however, genome reduction reveals genetic load that is masked by heterozygosity in tetraploids (Manrique-Carpintero et al., 2018). Genome sequencing of tetraploids show higher levels of deleterious alleles compared to diploids and increased missing genes in each haplotype of a tetraploid compared to the whole genome (Freire et al., 2021; Hoopes et al., 2022; Sun et al., 2022). The current study describes whole genome sequencing of nine legacy diploid potato clones that were selected from a breeding program for maturity under long-day conditions, pigmentation, disease resistance, tuber appearance and quality among other traits (De Jong et al., 2003; Jung et al., 2005, 2009; Li et al., 2005; De Koeyer et al., 2009; Tai et al., 2018). The history of these diploids include dihaploid extraction from tetraploids and introgression with wild *Solanum* relatives. The 10× Genomics Gem-Code technology was previously used for phased *de novo* assembly of pepper and potato RHv3 genomes (Hulse-Kemp et al., 2018; Zhou et al., 2020) and was utilized in the current study. Estimation of heterozygosity through k-mer analysis with read data showed the heterozygosity rate ranged between 1 and 9.04% among the nine non-inbred diploid clones. In comparison, the inbred clone, Solyntus, had a percent heterozygosity of 0.293% in the k-mer analysis (van Lieshout et al., 2020). The residual heterozygosity in Solyntus was attributed to a combination of spurious outcrossing and loss of homozygotes due to inbreeding depression. These results of the current study suggest that among the nine diploid clones, some have relatively high levels of homozygosity due to a pedigree with inbreeding of related individuals.

Both the DMv6.1 and RHv3 were used as references for SNP and CNV analysis of the nine diploid clones. The CNV variant calls analysis showed that there are greater differences between the clones when using different reference genomes than the differences found between the clones in the SNP analysis against different reference genomes. Copy number variation is abundant in potato and has a role in adaptation (Hardigan et al., 2017; Kyriakidou et al., 2020; Hoopes et al., 2022) and increasing the diversity and strength of the genomic tools for potato (Gálvez et al., 2017) will benefit the interpretation of variant calling results such as the ones of the current study, particularly for CNV. The SNP analysis also showed that clone 10908-06 had the greatest phylogenetic distance from the other diploids in the study. Moreover, the sequence data does not support H412-1 as a parent of 10908-06, but suggests that the pollen parent of 10908-06 may have been an unknown plant not included in the study. The SNP analysis in the current study was validated using GBS on a completely different set of plants, therefore any labeling error or mix-up for either clone occurred before the initiation of the current study. The tree showing the relationship of the nuclear genomes is similar to plastome and mitogenome phylogenies (Achakkagari et al., 2021a,b).

Diploid F_1_ hybrid breeding can provide a more efficient breeding system for combining alleles and introgressing beneficial genes from diploid wild *Solanum* relatives compared to the predominant tetraploid breeding for potato (Lindhout et al., 2011; Jansky et al., 2016; Zhang et al., 2021). An issue is that diploid potato has high levels of inbreeding depression (De Jong and Rowe, 1971; Zhang et al., 2019). Recent development of genome design strategies for F_1_ hybrid breeding in potato include analysis of deleterious alleles in diploid clones (Zhang et al., 2021). The current study expanded on this approach to examine percent heterozygous deleterious alleles across 5 Mb bins to visualize genome-wide patterns of deleterious alleles. Three deleterious alleles analyses with different protein reference databases were done for each of the diploid clones in the current study. The results show differences in patterns of deleterious alleles across the genome depending on which protein reference database is used. Others have previously observed that SNPs located where the protein reference database carries the derived (mutant) allele are less likely to be classified as deleterious than are SNPs located where the reference allele is ancestral (Simons et al., 2014). These results demonstrate that analyses using multiple protein reference databases can provide a more extensive identification of deleterious alleles. The present analysis of the nine non-inbred diploid potato clones showed variation in the levels of deleterious alleles and also in regions carrying high percentages of heterozygous deleterious alleles. There was a widespread distribution of deleterious alleles in the genomes including regions with both high and low gene density for all the clones. Diploid clones with the lowest levels of heterozygous deleterious alleles were W5281.2 and 12120-03, which also had low heterozygosity. KASP genotyping assays and sequence analysis indicated that clone H412-1 carried the chromosome 12 *Sli* mutation conferring self-compatibility. Several regions with heterozygous deleterious alleles on chromosome 12 were found, which concurred with the large segregation distortion previously described (Gardner et al., 2019; Eggers et al., 2021; Ma et al., 2021).

Genetic mapping data from a cross of 12120–03 × 07506–01, was used to identify heterozygous markers from these two clones with segregation distortion associated with loss of homozygous progeny in the next generation. This analysis led to identification of an NB-ARC disease resistance gene that carried two deleterious mutations and showed segregation distortion. The results suggest that this gene may have a critical role in the survival of potato plants. The NB-ARC family of disease resistance genes have an ATPase domain (van Ooijen et al., 2008) and are involved in resistance to PVX in potato (Rairdan and Moffett, 2006). Loss of progeny clones in the field during propagation of clones derived from the 12120–03 × 07506–01 cross was noted. Given the role of NB-ARC in disease resistance, it is likely that the impact of the homozygosity of deleterious alleles of the gene was during field propagation rather than lethality during zygote or gamete development.

Chromosome 5 carries the *StCDF1* locus which regulates photoperiod dependent tuberization in potato (Kloosterman et al., 2013). The *StCDF1.1* allele encodes a protein inducing tuberization that is functional under short-days but is degraded under long-days. *StCDF1* alleles *StCDF1.2*, *StCDF1.3*, *StCDF1.4* and the newly identified *StCDF1.5*^H412–1^ carry insertion mutations that disrupt the FKF domain responsible for photoperiod-regulated degradation of the protein. Plants that carry these alleles lose photoperiod regulation of tuberization and become day-neutral (Kloosterman et al., 2013; Gutaker et al., 2019). These variant alleles are critical for potato cultivation in the northern hemisphere under long days. The *StCDF1* locus was examined in the diploid clones. The *StCDF*^DW84–1457^ allele has an insertion in the same location as *StCDF1.2*, *StCDF1.3*, and *StCDF1.4*, however, unlike the insertions of these other alleles, the *StCDF*^DW84–1457^ variant encodes a full length StCDF1 protein with an FKF domain. Recent studies have shown that *StCDF1* is also regulated by the *StFLORE* lncRNA that is transcribed in the opposite direction and extends into the 3′ end of *StCDF1*, which includes the variant regions (Ramírez Gonzales et al., 2021). These results suggest that disruption of *StFLORE* may also be involved in regulation of *StCDF1*. The sequence analysis indicates that additional novel alleles of *StCDF1.1* were present. Most of the variants were single amino acid substitutions that did not disrupt the domain structure of the protein. Genome sequences of tetraploids also demonstrate variation in *StCDF1.1* alleles (Hoopes et al., 2022). All of the diploid clones in the study produced tubers under long day conditions, which suggests that they all carry a *StCDF1* allele that is functional under long days. Further studies will be needed to determine the functional differences in the novel *StCDF1* alleles identified in the diploid clones that enable tuberization under long days for these clones. Analysis of the wider genomic region surrounding the *StCDF1* locus in the nine diploid clones carried deletions, which is consistent with the high levels of sequence variation of the *StCDF1* gene observed in other studies (Hoopes et al., 2022). Additional higher depth and long read sequencing in the future will elucidate the functional nature of the deletions.

The study has shown widespread distribution of heterozygous deleterious alleles in nine of the legacy adapted diploids from the Agriculture and Agri-Food Canada (AAFC) germplasm collection. These heterozygous deleterious alleles can become unmasked with inbreeding, which is correlated with observations of severe inbreeding depression in the legacy diploid clones. Development of inbred lines for diploid F1 hybrid breeding will likely require several generations of selfing with selection against deleterious alleles, along with losses of beneficial alleles locked in repulsion. Use of the genome sequence information, especially concerning deleterious alleles, will be helpful for development of inbred lines.

## Data availability statement

The original contributions presented in this study are publicly available. This data can be found here: NCBI, PRJNA684565 and doi: 10.5061/dryad.2547d7wt8.

## Author contributions

SA performed an evaluation of assemblies, CNV analyses, SNP analyses, read depth, and deleterious allele analyses and contributed to writing Materials and methods and Results sections. MK performed the *de novo* genome assembly, contamination removal, scaffolding, CNV analyses, and heterozygosity analyses. KG contributed to the analysis of SNP variation of diploids. DD contributed to the cultivation of plant material and genetic analysis of diploids. HD developed the diploid clones and curated pedigree information. MS supervised the bioinformatics analysis and contributed to writing the manuscript. HT performed the segregation distortion analysis and was the lead writer of the manuscript. All authors contributed to the article and approved the submitted version.

## Footnotes

1
https://www.10xgenomics.com/

2
http://www.ncbi.nlm.nih.gov/tools/vecscreen/univec/

3
http://tree.bio.ed.ac.uk/software/figtree/

4
www.intertek.com/agriculture

5
https://en.vectorbuilder.com/tool/dna-secondary-structure.html
