# Supplementary material for: Genome sequencing of adapted diploid potato clones
Source: Front Plant Sci. 2022 Aug 8;13:954933. doi: 10.3389/fpls.2022.954933 (PMC9394749; doi:10.3389/fpls.2022.954933)
Supplement: Supplementary file 1 [file Data_Sheet_1.docx]

## Supplementary data


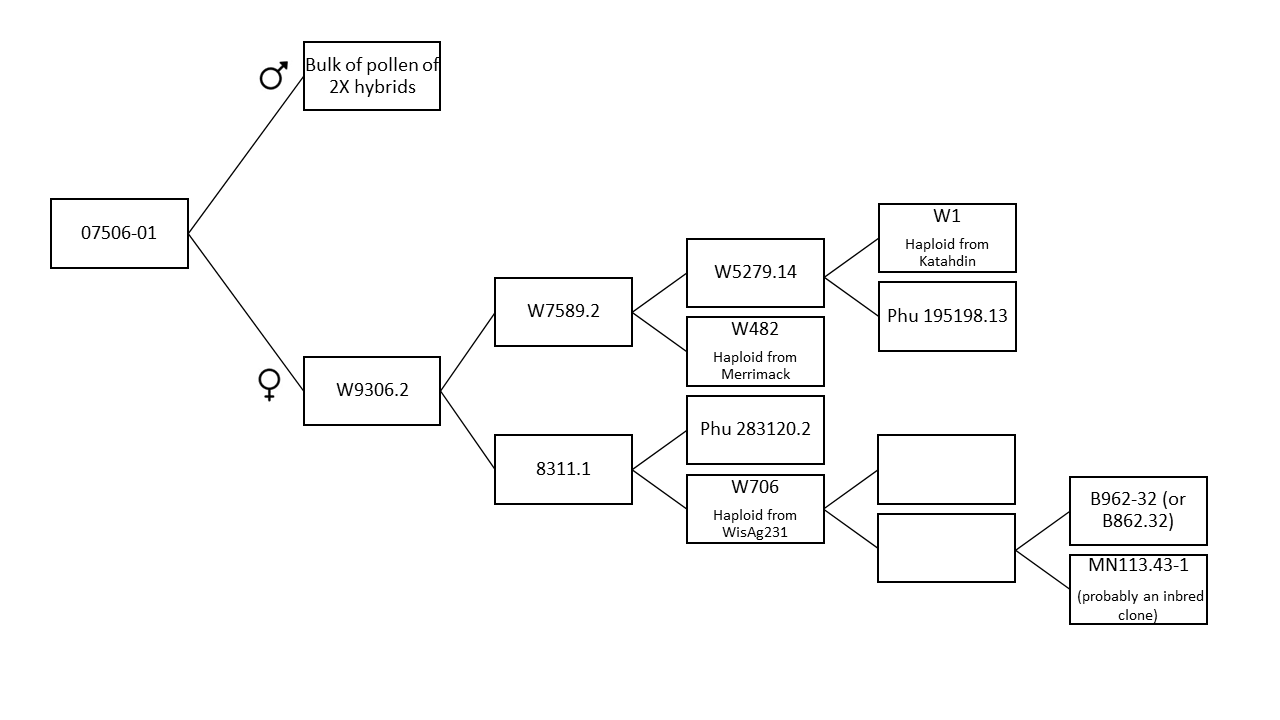

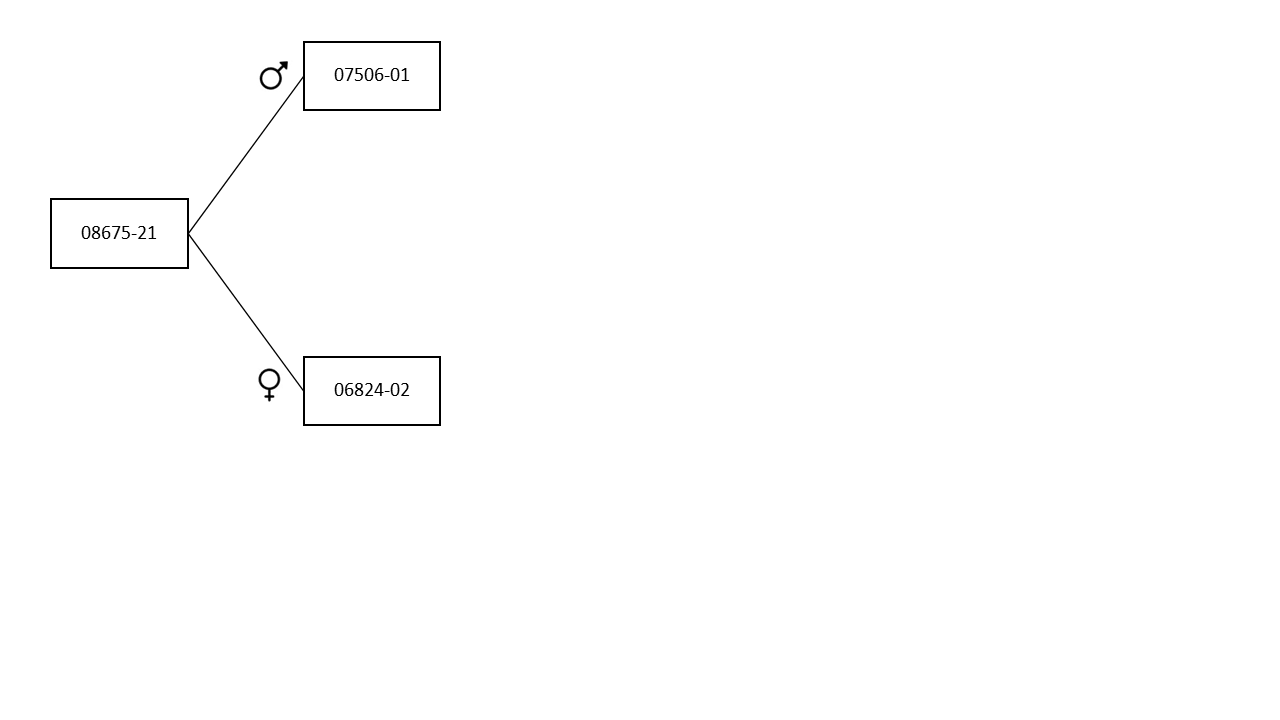

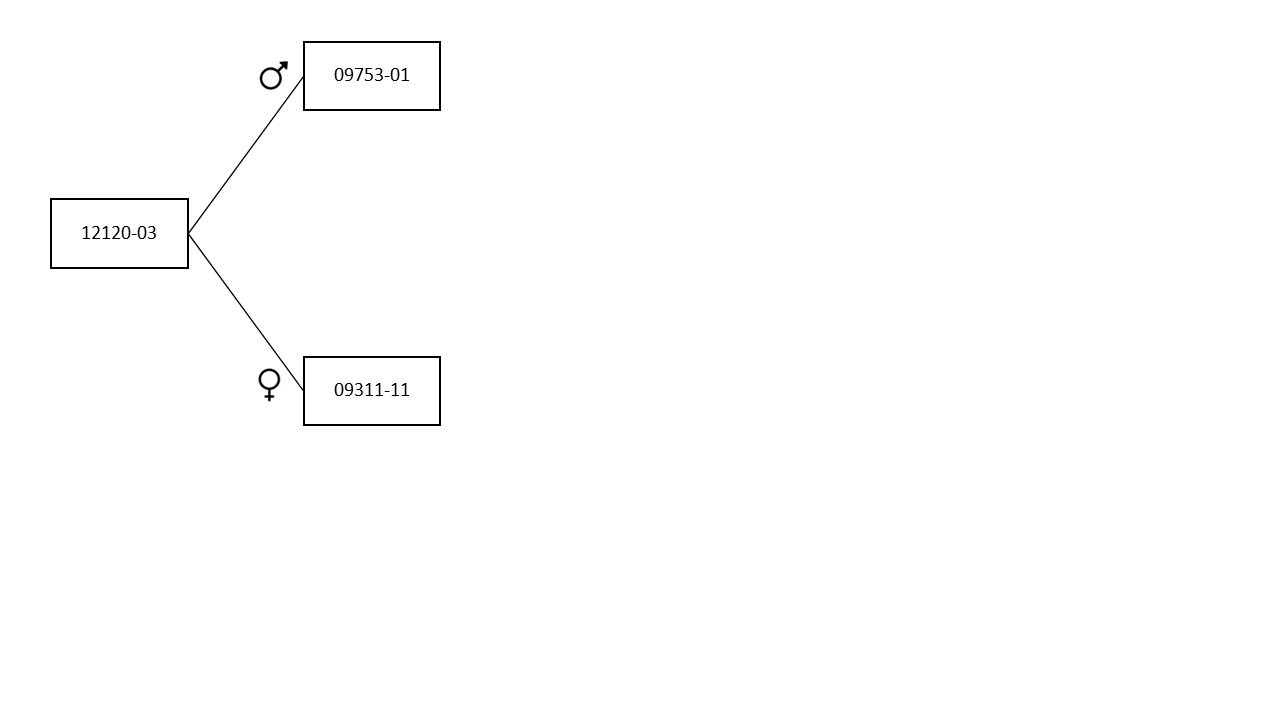

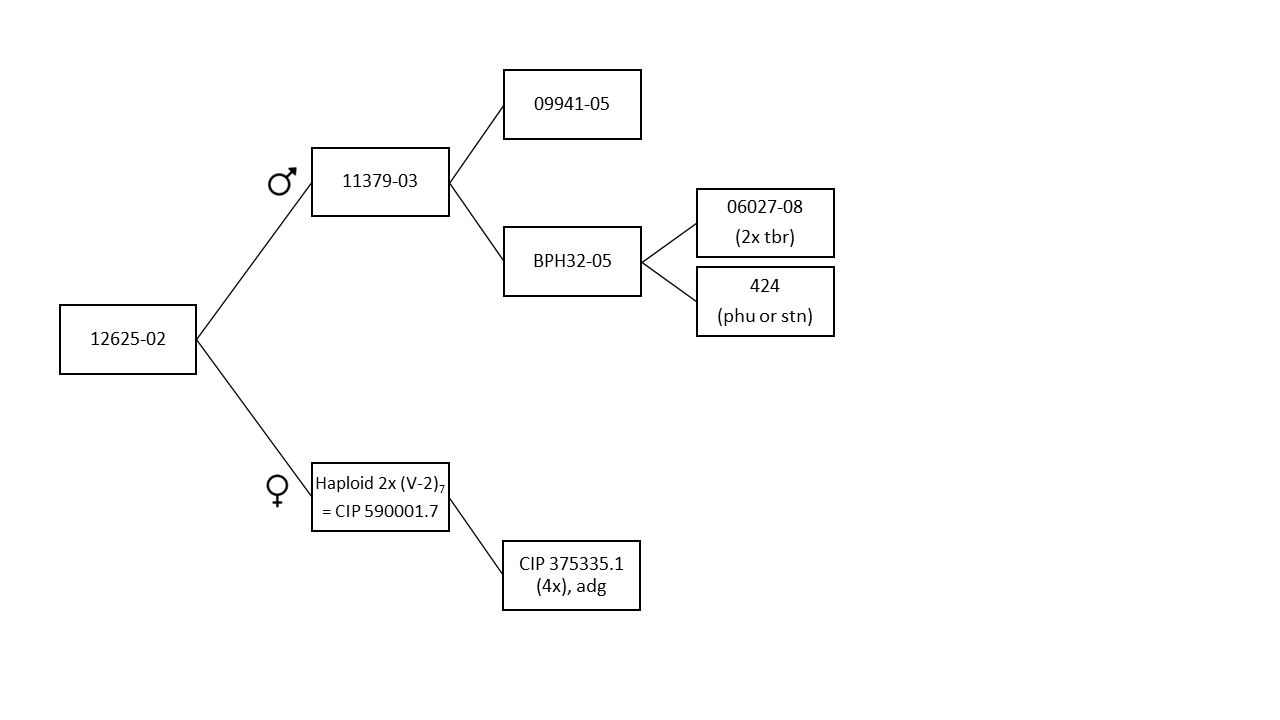

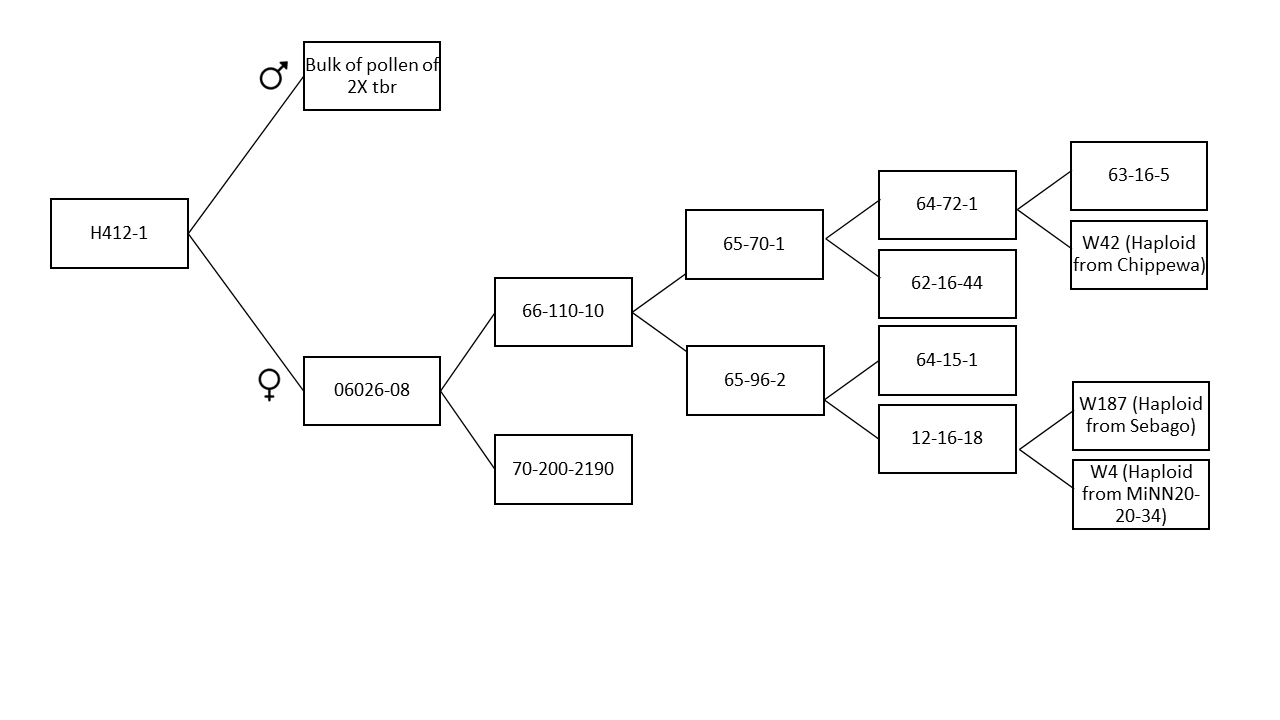

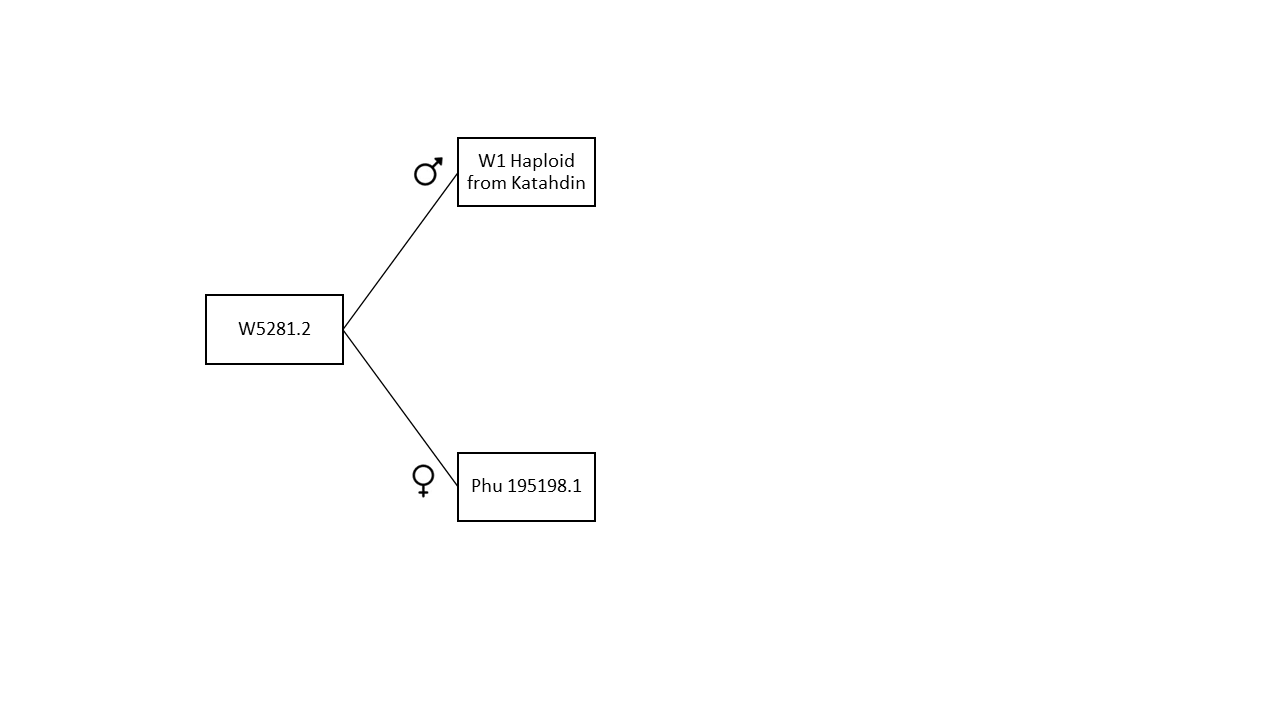

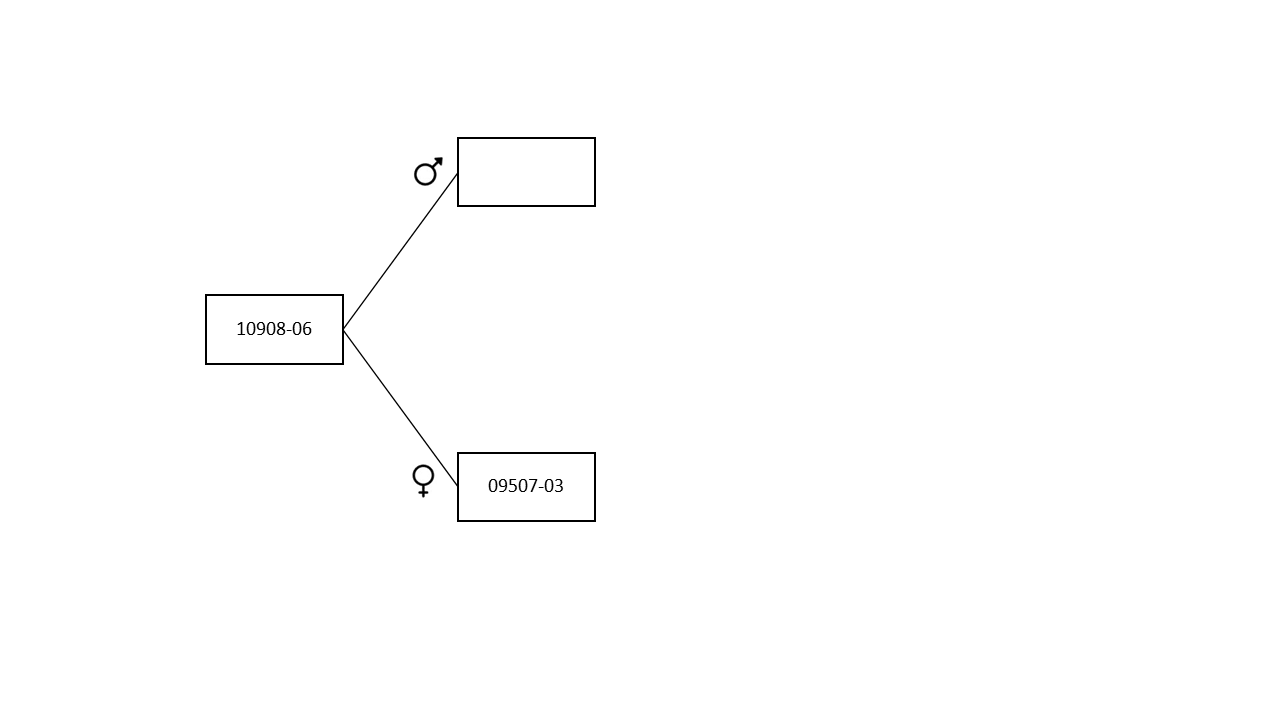

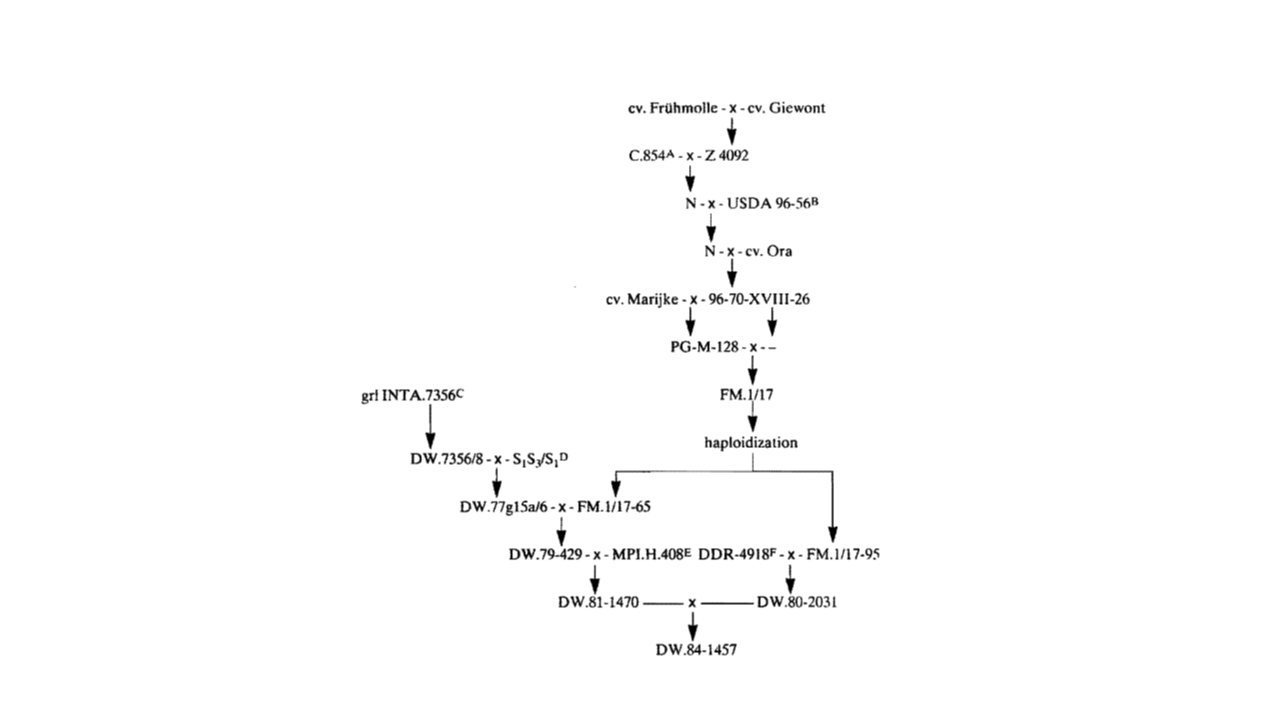


**Supplementary Figure 1** Pedigree charts of diploid clones. Boxes are blank where parent is unknown.

##


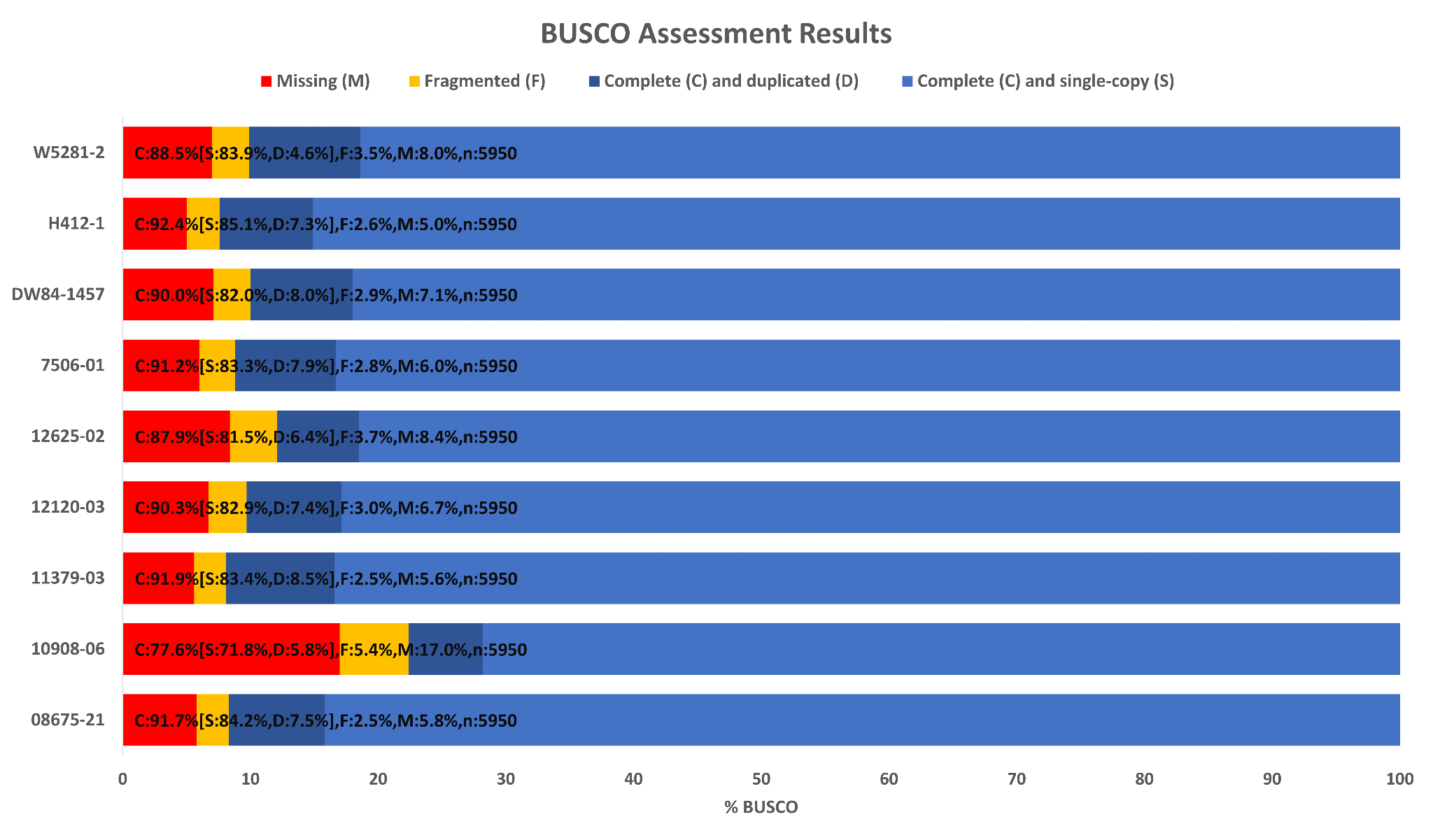


**Supplementary Figure 2**: BUSCO assessment results for each genome


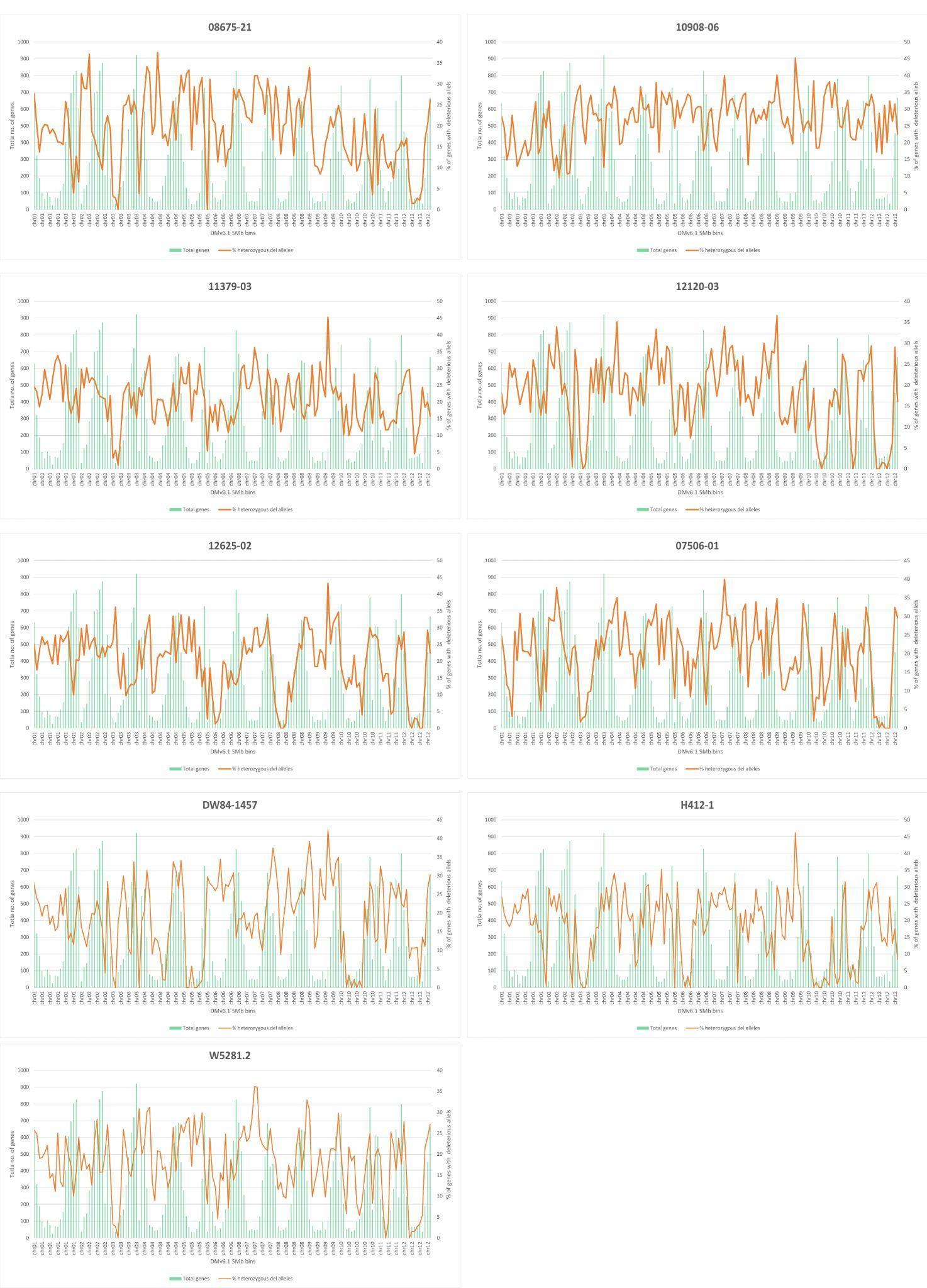


**Supplementary Figure 3**: Total gene density and % heterozygous deleterious alleles determined using the DMv6.1 reference. The x-axis indicates the 5 Mb bins of the genome and y-axis is the total number of genes on the left and the % genes with heterozygous deleterious alleles on the right.


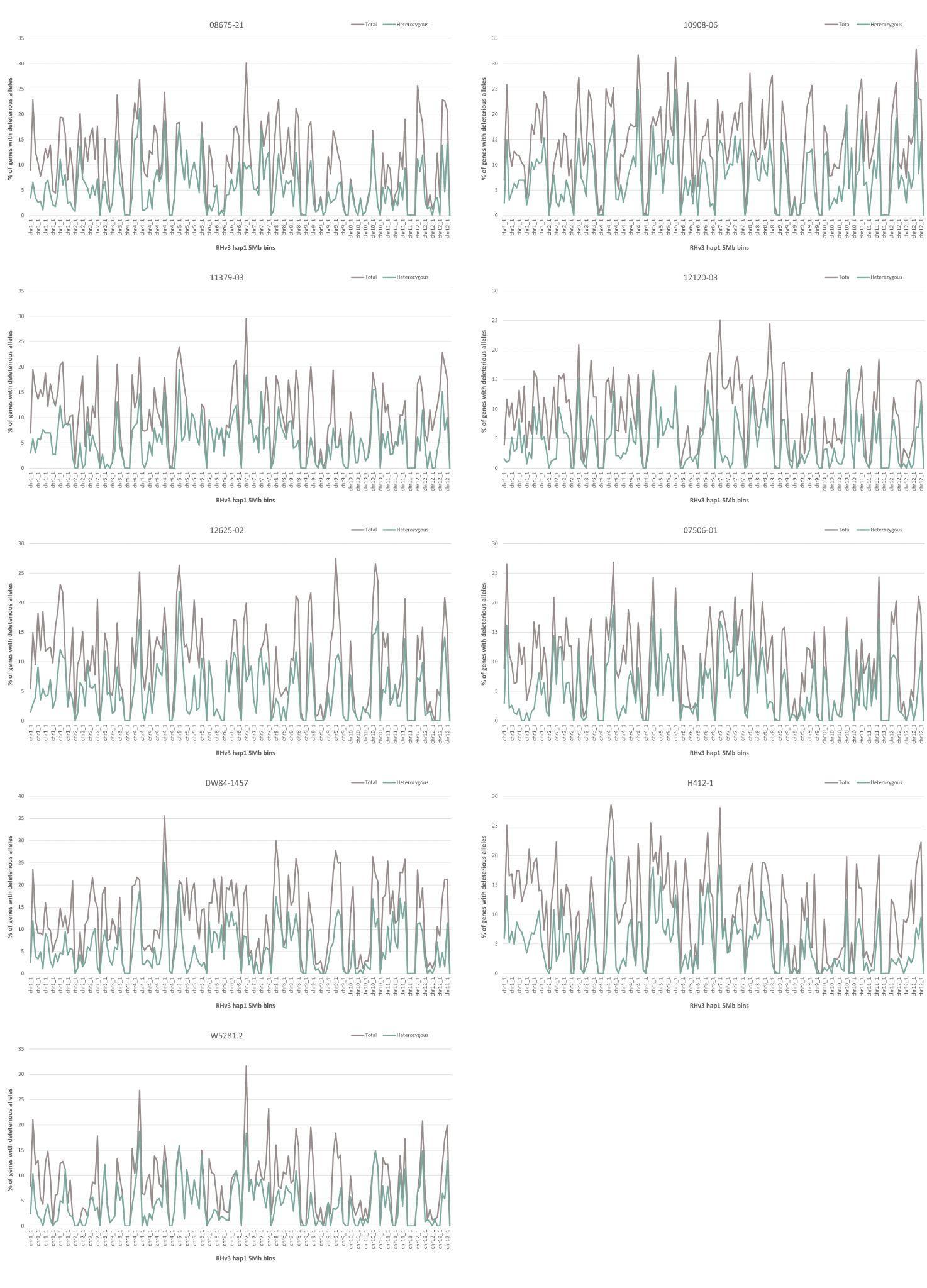


**Supplementary Figure 4**. Percentage of deleterious allele affected genes in 5 Mb bins of RHv3 haplotype 1.


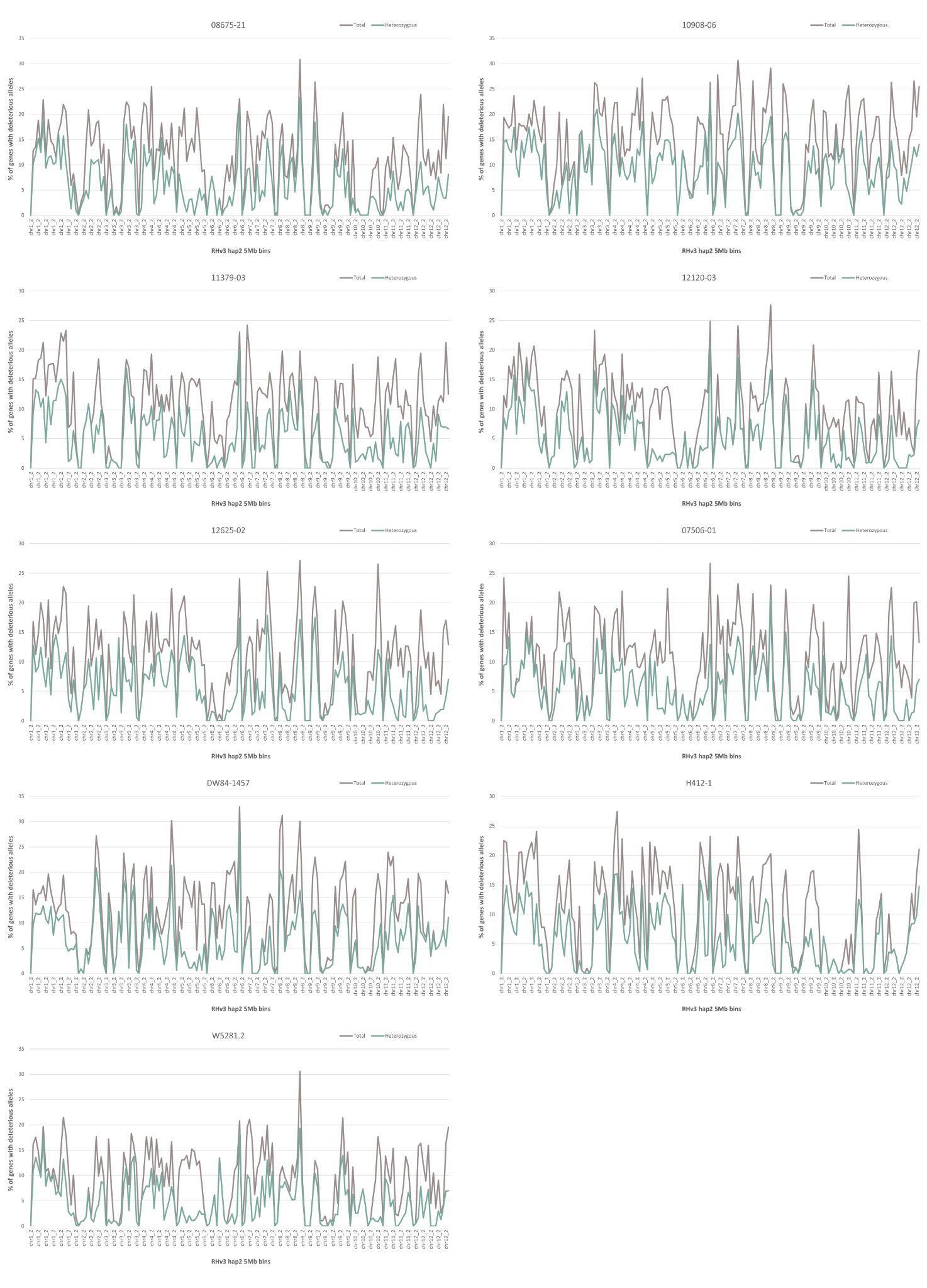


**Supplementary Figure 5**. Percentage of deleterious allele affected genes in 5 Mb bins of RHv3 haplotype 2.


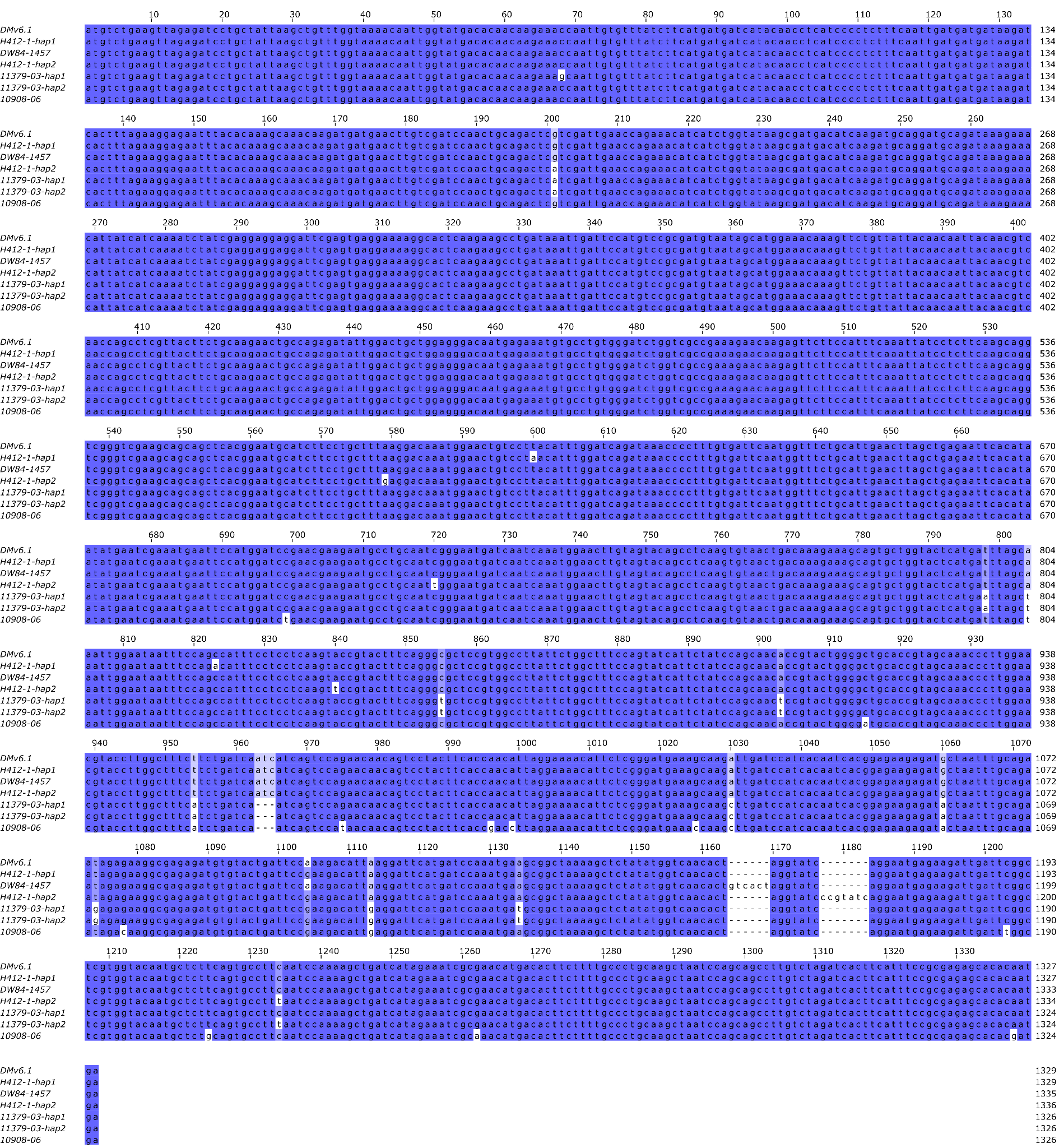


**Supplementary Figure 6**. Nucleotide sequence alignment of novel StCDF1 alleles from H412-1, DW84-1457, 11379-03 and 10908-06. H412-1 and 11379-03 had two different novel alleles in the two haplotypes, hap1 and hap2.


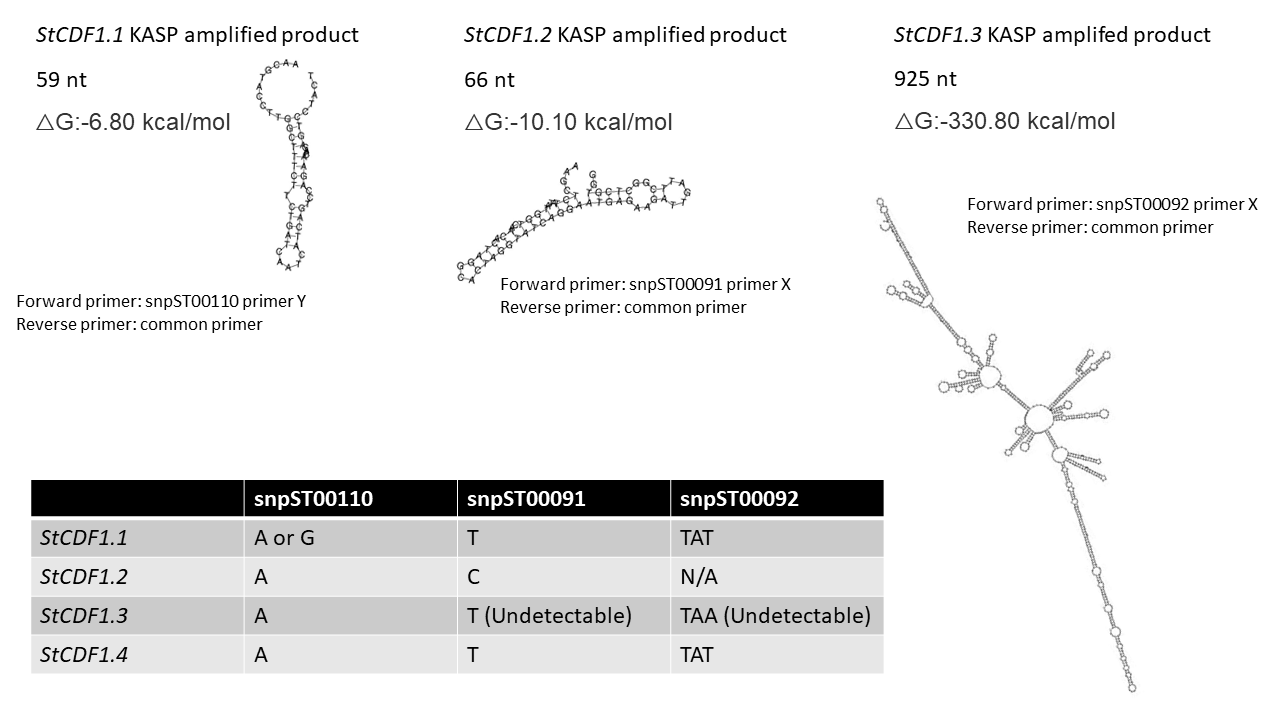


**Supplementary Figure 7**. Secondary structures of StCDF1.1, StCDF1.2, StCDF1.3 KASP assay PCR products.

**Supplementary Table 1**. Assembly statistics of the nine genomes after scaffolding

| **Genome** | **12625-02** | **H412-1** | **10908-06** | **08675-21** | **W5281-2** | **12120-03** | **07506-01** | **11379-03** | **DW84-1457** |
| --- | --- | --- | --- | --- | --- | --- | --- | --- | --- |
| Scaffold number | 101,184 | 74,884 | 179,805 | 70,604 | 73,187 | 89,052 | 84,615 | 78,624 | 73,260 |
| Assembly size (bp) | 827,062,754 | 901,443,132 | 712,637,036 | 912,493,466 | 868,622,197 | 843,392,895 | 897,343,495 | 948,106,575 | 882,591,920 |
| Contig N50 (bp) | 24,955 | 45,637 | 6,726 | 46,214 | 40,485 | 29,597 | 35,345 b | 42,465 | 39,082 |
| Scaffolds >=10 Kb | 18,879 | 17,431 | 13,823 | 17,568 | 17,596 | 18,672 | 18,941 | 18,594 | 18,800 |
| Largest contig (bp) | 482,791 | 1,129,528 | 236,590 | 1,313,652 | 757,208 | 599,577 | 581,055 | 1,107,497 | 700,324 |
| GC content (%) | 35.96 | 35.26 | 35.47 | 35.18 | 35.08 | 35.81 | 35.64 | 35.23 | 35.20 |

**Supplementary Table 2**. Number of SNPs detected in each genome when compared against DMv6.1

| **Genome** | **12625-02** | **H412-1** | **10908-06** | **08675-21** | **W5281-2** | **12120-03** | **07506-01** | **11379-03** | **DW84-1457** |
| --- | --- | --- | --- | --- | --- | --- | --- | --- | --- |
| Variant sites | 4,632,643 | 5,904,254 | 6,989,299 | 5,379,569 | 4,543,158 | 4,436,076 | 5,512,628 | 5,333,911 | 5,605,583 |
| Homozygous | 2,554,986 | 3,667,978 | 3,808,698 | 2,932,421 | 2,323,614 | 2,359,911 | 3,264,766 | 2,724,575 | 3,364,739 |
| Heterozygous | 2,065,273 | 2,221,861 | 3,156,042 | 2,434,053 | 2,209,940 | 2,066,076 | 2,232,786 | 2,595,344 | 2,228,553 |
| SNPs | 3,748,506 | 4,701,264 | 5,564,743 | 4,324,005 | 3,689,989 | 3,592,465 | 4,433,972 | 4,264,162 | 4,480,788 |
| MNPs | 622,045 | 829,571 | 1,026,637 | 722,448 | 571,430 | 570,698 | 746,107 | 746,495 | 766,222 |
| InDels | 383,589 | 555,280 | 595,577 | 490,512 | 409,483 | 396,853 | 489,273 | 481,270 | 529,207 |

**Supplementary Table 3**. Number of SNPs detected in each genome when compared against RHv3

| **Genome** | **12625-02** | **H412-1** | **10908-06** | **08675-21** | **W5281-2** | **12120-03** | **07506-01** | **11379-03** | **DW84-1457** |
| --- | --- | --- | --- | --- | --- | --- | --- | --- | --- |
| Variant sites | 4,767,641 | 5,142,063 | 7,223,705 | 4,940,066 | 4,108,381 | 4,148,462 | 4,832,991 | 4,922,709 | 5,872,718 |
| Homozygous | 3,394,308 | 3,631,171 | 4,678,855 | 3,292,726 | 2,678,919 | 2,756,824 | 3,237,249 | 3,306,288 | 4,071,432 |
| Heterozygous | 1,368,037 | 1,503,710 | 2,531,584 | 1,641,996 | 1,425,883 | 1,387,426 | 1,589,441 | 1,610,697 | 1,793,848 |
| SNPs | 3,917,886 | 4,128,127 | 5,821,790 | 4,015,020 | 3,382,015 | 3,415,485 | 3,940,720 | 4,006,302 | 4,747,908 |
| MNPs | 586,015 | 706,120 | 993,276 | 620,270 | 473,718 | 495,112 | 616,682 | 627,042 | 760,080 |
| InDels | 368,779 | 445,536 | 590,925 | 431,840 | 351,120 | 330,843 | 390,306 | 412,596 | 520,843 |

**Supplementary Table 4**. Percentage of heterozygosity in each genome obtained from jellyfish

| **Genome** | **12625-02** | **H412-1** | **10908-06** | **08675-21** | **W5281-2** | **12120-03** | **07506-01** | **11379-03** | **DW84-1457** |
| --- | --- | --- | --- | --- | --- | --- | --- | --- | --- |
| Heterozygosity (min) | 3.94 | 0 | 4.66 | 0 | 0 | 0 | 0 | 0 | 0 |
| Heterozygosity (max) | 9.04 | 2.08 | 5.15 | 1.6 | 5.17 | 1 | 1.42 | 8.48 | 4.11 |
| Average Read depth | 18 | 26 | 25 | 26 | 27 | 25 | 24 | 23 | 27 |
| Standard deviation | 227 | 99 | 247 | 143 | 115 | 103 | 92 | 115 | 122 |

**Supplementary Table 5**. Segregation distortion analysis from a cross of 12120-03 X 07506-01. The markers highlighted in yellow show linkage in repulsion shown in Figure 6.

| **marker** | **SolCAP** | **chr** | **position** | **parental genotype (12120-03x 07506-01)** | **exp hom1** | **exp het** | **exp hom2** | **obs hom1** | **obs het** | **obs hom2** | **chi-sq p-value** | **Bonferroni adjusted p-value** |
| --- | --- | --- | --- | --- | --- | --- | --- | --- | --- | --- | --- | --- |
| SNP_3218 | solcap_snp_c2_21098 | chr01 | 2617286 | <hkxhk> | 22.5 | 45 | 22.5 | 20 | 50 | 18 | 0.421626 | 1 |
| SNP_7397 | solcap_snp_c2_55012 | chr01 | 5654482 | <hkxhk> | 22.5 | 45 | 22.5 | 27 | 46 | 14 | 0.124157 | 1 |
| SNP_7576 | solcap_snp_c2_56125 | chr01 | 6011953 | <hkxhk> | 22.5 | 45 | 22.5 | 14 | 43 | 31 | 0.036632 | 1 |
| SNP_5917 | solcap_snp_c2_45058 | chr01 | 6065326 | <hkxhk> | 22.5 | 45 | 22.5 | 30 | 42 | 15 | 0.071509 | 1 |
| SNP_5921 | solcap_snp_c2_45071 | chr01 | 6102951 | <hkxhk> | 22.5 | 45 | 22.5 | 31 | 42 | 15 | 0.049787 | 1 |
| SNP_3430 | solcap_snp_c2_2334 | chr01 | 9770457 | <hkxhk> | 22.5 | 45 | 22.5 | 30 | 47 | 12 | 0.022802 | 1 |
| SNP_7610 | solcap_snp_c2_56402 | chr01 | 10364572 | <hkxhk> | 22.5 | 45 | 22.5 | 31 | 47 | 10 | 0.005429 | 1 |
| SNP_6466 | solcap_snp_c2_49042 | chr01 | 10922747 | <hkxhk> | 22.5 | 45 | 22.5 | 33 | 49 | 7 | 0.000318 | 0.335808 |
| SNP_7137 | solcap_snp_c2_53521 | chr01 | 12643852 | <hkxhk> | 22.5 | 45 | 22.5 | 33 | 51 | 5 | 5.78E-05 | 0.061248 |
| SNP_5455 | solcap_snp_c2_40955 | chr01 | 65187446 | <hkxhk> | 22.5 | 45 | 22.5 | 3 | 55 | 30 | 1.61E-05 | 1 |
| SNP_5456 | solcap_snp_c2_40956 | chr01 | 65334579 | <hkxhk> | 22.5 | 45 | 22.5 | 30 | 55 | 3 | 1.61E-05 | 1 |
| SNP_1398 | solcap_snp_c1_4706 | chr01 | 67245991 | <hkxhk> | 22.5 | 45 | 22.5 | 28 | 56 | 4 | 5.44E-05 | 0.057024 |
| SNP_2525 | solcap_snp_c2_14495 | chr01 | 67249102 | <hkxhk> | 22.5 | 45 | 22.5 | 4 | 56 | 28 | 5.44E-05 | 0.057024 |
| SNP_1642 | solcap_snp_c1_6518 | chr01 | 68869103 | <hkxhk> | 22.5 | 45 | 22.5 | 31 | 45 | 13 | 0.026093 | 1 |
| SNP_7006 | solcap_snp_c2_52484 | chr01 | 69319152 | <hkxhk> | 22.5 | 45 | 22.5 | 30 | 45 | 14 | 0.056021 | 1 |
| SNP_6787 | solcap_snp_c2_51057 | chr01 | 70110356 | <hkxhk> | 22.5 | 45 | 22.5 | 28 | 46 | 14 | 0.098452 | 1 |
| SNP_0917 | solcap_snp_c1_16178 | chr01 | 70272823 | <hkxhk> | 22.5 | 45 | 22.5 | 10 | 44 | 33 | 0.002274 | 1 |
| SNP_5352 | solcap_snp_c2_39834 | chr01 | 70588122 | <hkxhk> | 22.5 | 45 | 22.5 | 33 | 44 | 10 | 0.002274 | 1 |
| SNP_2858 | solcap_snp_c2_17592 | chr01 | 71013750 | <hkxhk> | 22.5 | 45 | 22.5 | 33 | 43 | 12 | 0.006512 | 1 |
| SNP_2320 | solcap_snp_c2_12076 | chr01 | 72181076 | <hkxhk> | 22.5 | 45 | 22.5 | 13 | 46 | 29 | 0.049787 | 1 |
| SNP_1392 | solcap_snp_c1_4622 | chr01 | 75291402 | <hkxhk> | 22.5 | 45 | 22.5 | 25 | 45 | 18 | 0.560152 | 1 |
| SNP_2508 | solcap_snp_c2_14365 | chr01 | 75614887 | <hkxhk> | 22.5 | 45 | 22.5 | 18 | 45 | 25 | 0.560152 | 1 |
| SNP_8061 | solcap_snp_c2_7193 | chr01 | 80006781 | <hkxhk> | 22.5 | 45 | 22.5 | 17 | 41 | 31 | 0.08395 | 1 |
| SNP_1117 | solcap_snp_c1_2520 | chr01 | 80101013 | <hkxhk> | 22.5 | 45 | 22.5 | 18 | 50 | 19 | 0.374277 | 1 |
| SNP_8067 | solcap_snp_c2_7241 | chr01 | 80280265 | <hkxhk> | 22.5 | 45 | 22.5 | 19 | 49 | 18 | 0.427912 | 1 |
| SNP_1119 | solcap_snp_c1_2535 | chr01 | 80381278 | <hkxhk> | 22.5 | 45 | 22.5 | 31 | 41 | 17 | 0.08395 | 1 |
| SNP_1218 | solcap_snp_c1_3255 | chr01 | 82294166 | <hkxhk> | 22.5 | 45 | 22.5 | 18 | 39 | 30 | 0.119948 | 1 |
| SNP_1217 | solcap_snp_c1_3241 | chr01 | 82422618 | <hkxhk> | 22.5 | 45 | 22.5 | 18 | 40 | 29 | 0.187793 | 1 |
| SNP_0967 | solcap_snp_c1_1645 | chr01 | 83396737 | <hkxhk> | 22.5 | 45 | 22.5 | 31 | 39 | 17 | 0.06598 | 1 |
| SNP_6471 | solcap_snp_c2_4906 | chr01 | 83813441 | <hkxhk> | 22.5 | 45 | 22.5 | 32 | 38 | 17 | 0.037567 | 1 |
| SNP_1022 | solcap_snp_c1_1737 | chr01 | 83995128 | <hkxhk> | 22.5 | 45 | 22.5 | 32 | 37 | 18 | 0.03979 | 1 |
| SNP_6131 | solcap_snp_c2_4664 | chr01 | 84438658 | <hkxhk> | 22.5 | 45 | 22.5 | 31 | 38 | 18 | 0.071509 | 1 |
| SNP_6190 | solcap_snp_c2_4701 | chr01 | 84556055 | <hkxhk> | 22.5 | 45 | 22.5 | 18 | 40 | 31 | 0.094994 | 1 |
| SNP_6193 | solcap_snp_c2_4705 | chr01 | 84583451 | <hkxhk> | 22.5 | 45 | 22.5 | 31 | 39 | 17 | 0.06598 | 1 |
| SNP_1419 | solcap_snp_c1_4796 | chr01 | 86617312 | <hkxhk> | 22.5 | 45 | 22.5 | 28 | 36 | 23 | 0.20588 | 1 |
| SNP_2583 | solcap_snp_c2_14841 | chr01 | 87277528 | <hkxhk> | 22.5 | 45 | 22.5 | 28 | 34 | 25 | 0.113249 | 1 |
| SNP_4252 | solcap_snp_c2_31041 | chr01 | 88066153 | <hkxhk> | 22.5 | 45 | 22.5 | 27 | 36 | 24 | 0.247448 | 1 |
| SNP_4250 | solcap_snp_c2_30973 | chr01 | 88257433 | <hkxhk> | 22.5 | 45 | 22.5 | 29 | 34 | 24 | 0.094225 | 1 |
| SNP_0865 | solcap_snp_c1_15874 | chr02 | 9210579 | <hkxhk> | 22.5 | 45 | 22.5 | 30 | 39 | 19 | 0.143247 | 1 |
| SNP_5475 | solcap_snp_c2_41108 | chr02 | 17264294 | <hkxhk> | 22.5 | 45 | 22.5 | 18 | 40 | 30 | 0.135335 | 1 |
| SNP_0885 | solcap_snp_c1_15974 | chr02 | 17740155 | <hkxhk> | 22.5 | 45 | 22.5 | 30 | 40 | 18 | 0.135335 | 1 |
| SNP_1526 | solcap_snp_c1_5732 | chr02 | 19588567 | <hkxhk> | 22.5 | 45 | 22.5 | 29 | 39 | 19 | 0.198902 | 1 |
| SNP_5400 | solcap_snp_c2_40336 | chr02 | 20320425 | <hkxhk> | 22.5 | 45 | 22.5 | 28 | 40 | 19 | 0.297409 | 1 |
| SNP_0206 | solcap_snp_c1_11344 | chr02 | 20827869 | <hkxhk> | 22.5 | 45 | 22.5 | 29 | 38 | 20 | 0.196628 | 1 |
| SNP_2072 | solcap_snp_c1_9719 | chr02 | 21788128 | <hkxhk> | 22.5 | 45 | 22.5 | 28 | 38 | 21 | 0.284045 | 1 |
| SNP_0168 | solcap_snp_c1_11122 | chr02 | 22851833 | <hkxhk> | 22.5 | 45 | 22.5 | 27 | 38 | 22 | 0.374277 | 1 |
| SNP_1668 | solcap_snp_c1_6841 | chr02 | 24058958 | <hkxhk> | 22.5 | 45 | 22.5 | 19 | 49 | 20 | 0.560152 | 1 |
| SNP_3267 | solcap_snp_c2_21722 | chr02 | 24233734 | <hkxhk> | 22.5 | 45 | 22.5 | 19 | 50 | 20 | 0.501069 | 1 |
| SNP_3263 | solcap_snp_c2_21682 | chr02 | 24387720 | <hkxhk> | 22.5 | 45 | 22.5 | 19 | 47 | 21 | 0.720661 | 1 |
| SNP_5954 | solcap_snp_c2_45307 | chr02 | 25888156 | <hkxhk> | 22.5 | 45 | 22.5 | 21 | 49 | 18 | 0.511476 | 1 |
| SNP_5955 | solcap_snp_c2_45308 | chr02 | 25888240 | <hkxhk> | 22.5 | 45 | 22.5 | 22 | 42 | 24 | 0.872525 | 1 |
| SNP_0377 | solcap_snp_c1_12329 | chr02 | 26364945 | <hkxhk> | 22.5 | 45 | 22.5 | 18 | 49 | 20 | 0.476456 | 1 |
| SNP_3080 | solcap_snp_c2_19692 | chr02 | 27348959 | <hkxhk> | 22.5 | 45 | 22.5 | 23 | 42 | 23 | 0.9131 | 1 |
| SNP_1454 | solcap_snp_c1_5088 | chr02 | 27433808 | <hkxhk> | 22.5 | 45 | 22.5 | 23 | 41 | 23 | 0.866166 | 1 |
| SNP_5249 | solcap_snp_c2_39155 | chr02 | 27899170 | <hkxhk> | 22.5 | 45 | 22.5 | 19 | 49 | 20 | 0.560152 | 1 |
| SNP_0570 | solcap_snp_c1_13929 | chr02 | 28460905 | <hkxhk> | 22.5 | 45 | 22.5 | 25 | 40 | 22 | 0.680411 | 1 |
| SNP_6166 | solcap_snp_c2_46908 | chr02 | 28461650 | <hkxhk> | 22.5 | 45 | 22.5 | 22 | 40 | 24 | 0.774286 | 1 |
| SNP_0566 | solcap_snp_c1_13911 | chr02 | 28554779 | <hkxhk> | 22.5 | 45 | 22.5 | 18 | 50 | 20 | 0.421626 | 1 |
| SNP_6160 | solcap_snp_c2_46898 | chr02 | 28682663 | <hkxhk> | 22.5 | 45 | 22.5 | 18 | 49 | 21 | 0.511476 | 1 |
| SNP_3395 | solcap_snp_c2_23139 | chr02 | 29015258 | <hkxhk> | 22.5 | 45 | 22.5 | 23 | 43 | 21 | 0.94959 | 1 |
| SNP_5520 | solcap_snp_c2_41541 | chr02 | 30008586 | <hkxhk> | 22.5 | 45 | 22.5 | 23 | 40 | 23 | 0.811149 | 1 |
| SNP_0483 | solcap_snp_c1_13236 | chr02 | 30080610 | <hkxhk> | 22.5 | 45 | 22.5 | 24 | 40 | 23 | 0.745944 | 1 |
| SNP_5899 | solcap_snp_c2_44982 | chr02 | 30298041 | <hkxhk> | 22.5 | 45 | 22.5 | 20 | 42 | 25 | 0.712425 | 1 |
| SNP_6425 | solcap_snp_c2_48784 | chr02 | 31811846 | <hkxhk> | 22.5 | 45 | 22.5 | 20 | 43 | 25 | 0.735784 | 1 |
| SNP_0370 | solcap_snp_c1_12257 | chr02 | 32346953 | <hkxhk> | 22.5 | 45 | 22.5 | 19 | 44 | 24 | 0.745944 | 1 |
| SNP_6714 | solcap_snp_c2_50412 | chr02 | 33645025 | <hkxhk> | 22.5 | 45 | 22.5 | 19 | 41 | 28 | 0.324652 | 1 |
| SNP_2888 | solcap_snp_c2_17858 | chr02 | 34602898 | <hkxhk> | 22.5 | 45 | 22.5 | 18 | 45 | 25 | 0.560152 | 1 |
| SNP_5579 | solcap_snp_c2_42244 | chr02 | 35355594 | <hkxhk> | 22.5 | 45 | 22.5 | 19 | 44 | 24 | 0.745944 | 1 |
| SNP_0915 | solcap_snp_c1_16171 | chr02 | 35468365 | <hkxhk> | 22.5 | 45 | 22.5 | 21 | 45 | 21 | 0.94959 | 1 |
| SNP_7089 | solcap_snp_c2_53035 | chr02 | 36571884 | <hkxhk> | 22.5 | 45 | 22.5 | 22 | 50 | 16 | 0.29309 | 1 |
| SNP_7087 | solcap_snp_c2_53033 | chr02 | 36572054 | <hkxhk> | 22.5 | 45 | 22.5 | 26 | 40 | 21 | 0.56611 | 1 |
| SNP_3676 | solcap_snp_c2_25897 | chr02 | 37987353 | <hkxhk> | 22.5 | 45 | 22.5 | 21 | 35 | 32 | 0.040118 | 1 |
| SNP_3620 | solcap_snp_c2_25179 | chr02 | 38138741 | <hkxhk> | 22.5 | 45 | 22.5 | 32 | 34 | 21 | 0.031256 | 1 |
| SNP_8090 | solcap_snp_c2_7543 | chr02 | 38924917 | <hkxhk> | 22.5 | 45 | 22.5 | 36 | 34 | 20 | 0.003952 | 1 |
| SNP_0984 | solcap_snp_c1_16540 | chr02 | 40026564 | <hkxhk> | 22.5 | 45 | 22.5 | 12 | 59 | 17 | 0.004526 | 1 |
| SNP_3371 | solcap_snp_c2_22939 | chr02 | 40178088 | <hkxhk> | 22.5 | 45 | 22.5 | 12 | 61 | 14 | 0.000836 | 0.882816 |
| SNP_2636 | solcap_snp_c2_15070 | chr02 | 43229397 | <hkxhk> | 22.5 | 45 | 22.5 | 15 | 63 | 10 | 0.000205 | 0.21648 |
| SNP_0853 | solcap_snp_c1_15839 | chr03 | 2478421 | <hkxhk> | 22.5 | 45 | 22.5 | 18 | 40 | 29 | 0.187793 | 1 |
| SNP_1489 | solcap_snp_c1_5374 | chr03 | 3570186 | <hkxhk> | 22.5 | 45 | 22.5 | 18 | 38 | 30 | 0.104788 | 1 |
| SNP_5830 | solcap_snp_c2_44361 | chr03 | 4409064 | <hkxhk> | 22.5 | 45 | 22.5 | 18 | 37 | 31 | 0.060668 | 1 |
| SNP_0358 | solcap_snp_c1_12182 | chr03 | 4476856 | <hkxhk> | 22.5 | 45 | 22.5 | 17 | 38 | 32 | 0.037567 | 1 |
| SNP_7004 | solcap_snp_c2_52467 | chr03 | 4623495 | <hkxhk> | 22.5 | 45 | 22.5 | 32 | 38 | 17 | 0.037567 | 1 |
| SNP_1973 | solcap_snp_c1_8970 | chr03 | 5213394 | <hkxhk> | 22.5 | 45 | 22.5 | 17 | 36 | 33 | 0.016305 | 1 |
| SNP_7347 | solcap_snp_c2_54674 | chr03 | 7892640 | <hkxhk> | 22.5 | 45 | 22.5 | 18 | 36 | 33 | 0.020665 | 1 |
| SNP_0744 | solcap_snp_c1_15204 | chr03 | 9003615 | <hkxhk> | 22.5 | 45 | 22.5 | 19 | 35 | 33 | 0.019964 | 1 |
| SNP_0766 | solcap_snp_c1_15343 | chr03 | 37209342 | <hkxhk> | 22.5 | 45 | 22.5 | 14 | 35 | 40 | 6.61E-05 | 1 |
| SNP_1708 | solcap_snp_c1_7133 | chr03 | 37327657 | <hkxhk> | 22.5 | 45 | 22.5 | 14 | 36 | 39 | 0.000175 | 0.1848 |
| SNP_0205 | solcap_snp_c1_11339 | chr03 | 39584039 | <hkxhk> | 22.5 | 45 | 22.5 | 38 | 37 | 13 | 0.00027 | 0.28512 |
| SNP_3614 | solcap_snp_c2_24983 | chr03 | 40738583 | <hkxhk> | 22.5 | 45 | 22.5 | 35 | 39 | 14 | 0.003774 | 1 |
| SNP_7748 | solcap_snp_c2_57349 | chr03 | 40822476 | <hkxhk> | 22.5 | 45 | 22.5 | 34 | 40 | 13 | 0.004745 | 1 |
| SNP_1670 | solcap_snp_c1_6853 | chr03 | 41167892 | <hkxhk> | 22.5 | 45 | 22.5 | 14 | 40 | 34 | 0.007379 | 1 |
| SNP_0553 | solcap_snp_c1_13847 | chr03 | 42422747 | <hkxhk> | 22.5 | 45 | 22.5 | 33 | 41 | 14 | 0.013476 | 1 |
| SNP_2489 | solcap_snp_c2_13811 | chr03 | 42546228 | <hkxhk> | 22.5 | 45 | 22.5 | 15 | 41 | 32 | 0.030542 | 1 |
| SNP_7406 | solcap_snp_c2_55072 | chr03 | 44717428 | <hkxhk> | 22.5 | 45 | 22.5 | 33 | 41 | 14 | 0.013476 | 1 |
| SNP_7163 | solcap_snp_c2_53699 | chr03 | 44787719 | <hkxhk> | 22.5 | 45 | 22.5 | 31 | 43 | 14 | 0.036632 | 1 |
| SNP_4103 | solcap_snp_c2_29639 | chr03 | 45705706 | <hkxhk> | 22.5 | 45 | 22.5 | 13 | 47 | 27 | 0.079302 | 1 |
| SNP_7426 | solcap_snp_c2_55279 | chr03 | 47347920 | <hkxhk> | 22.5 | 45 | 22.5 | 31 | 43 | 14 | 0.036632 | 1 |
| SNP_2705 | solcap_snp_c2_1616 | chr03 | 49230263 | <hkxhk> | 22.5 | 45 | 22.5 | 27 | 41 | 19 | 0.415069 | 1 |
| SNP_2645 | solcap_snp_c2_1533 | chr03 | 49568103 | <hkxhk> | 22.5 | 45 | 22.5 | 13 | 49 | 26 | 0.083023 | 1 |
| SNP_2503 | solcap_snp_c2_14064 | chr03 | 51398803 | <hkxhk> | 22.5 | 45 | 22.5 | 10 | 49 | 27 | 0.01503 | 1 |
| SNP_1384 | solcap_snp_c1_4576 | chr03 | 51846728 | <hkxhk> | 22.5 | 45 | 22.5 | 28 | 39 | 20 | 0.300848 | 1 |
| SNP_1538 | solcap_snp_c1_5796 | chr03 | 52125132 | <hkxhk> | 22.5 | 45 | 22.5 | 9 | 51 | 26 | 0.007837 | 1 |
| SNP_2927 | solcap_snp_c2_18269 | chr03 | 53804623 | <hkxhk> | 22.5 | 45 | 22.5 | 9 | 49 | 29 | 0.005026 | 1 |
| SNP_2938 | solcap_snp_c2_18457 | chr03 | 54323415 | <hkxhk> | 22.5 | 45 | 22.5 | 9 | 50 | 29 | 0.004683 | 1 |
| SNP_3726 | solcap_snp_c2_26454 | chr03 | 54983223 | <hkxhk> | 22.5 | 45 | 22.5 | 9 | 51 | 27 | 0.006622 | 1 |
| SNP_3715 | solcap_snp_c2_26320 | chr03 | 55428180 | <hkxhk> | 22.5 | 45 | 22.5 | 9 | 53 | 25 | 0.006622 | 1 |
| SNP_4379 | solcap_snp_c2_322 | chr03 | 56043739 | <hkxhk> | 22.5 | 45 | 22.5 | 25 | 53 | 9 | 0.006622 | 1 |
| SNP_3566 | solcap_snp_c2_245 | chr03 | 56334740 | <hkxhk> | 22.5 | 45 | 22.5 | 10 | 54 | 25 | 0.010502 | 1 |
| SNP_3458 | solcap_snp_c2_23511 | chr04 | 802773 | <hkxhk> | 22.5 | 45 | 22.5 | 31 | 47 | 10 | 0.005429 | 1 |
| SNP_3464 | solcap_snp_c2_23602 | chr04 | 1365140 | <hkxhk> | 22.5 | 45 | 22.5 | 30 | 49 | 8 | 0.001913 | 1 |
| SNP_7288 | solcap_snp_c2_54381 | chr04 | 1559945 | <hkxhk> | 22.5 | 45 | 22.5 | 8 | 50 | 32 | 0.000953 | 1 |
| SNP_7110 | solcap_snp_c2_53206 | chr04 | 2605338 | <hkxhk> | 22.5 | 45 | 22.5 | 33 | 52 | 3 | 8.45E-06 | 1 |
| SNP_2258 | solcap_snp_c2_11487 | chr04 | 7749352 | <hkxhk> | 22.5 | 45 | 22.5 | 24 | 36 | 26 | 0.305426 | 1 |
| SNP_2255 | solcap_snp_c2_11432 | chr04 | 7884179 | <hkxhk> | 22.5 | 45 | 22.5 | 25 | 36 | 26 | 0.271281 | 1 |
| SNP_6436 | solcap_snp_c2_48859 | chr04 | 8265063 | <hkxhk> | 22.5 | 45 | 22.5 | 25 | 37 | 27 | 0.270093 | 1 |
| SNP_1888 | solcap_snp_c1_8353 | chr04 | 10469246 | <hkxhk> | 22.5 | 45 | 22.5 | 23 | 39 | 26 | 0.511476 | 1 |
| SNP_3786 | solcap_snp_c2_26843 | chr04 | 11028721 | <hkxhk> | 22.5 | 45 | 22.5 | 5 | 51 | 32 | 8.29E-05 | 1 |
| SNP_7237 | solcap_snp_c2_54083 | chr04 | 12263926 | <hkxhk> | 22.5 | 45 | 22.5 | 24 | 41 | 23 | 0.805808 | 1 |
| SNP_7592 | solcap_snp_c2_56255 | chr04 | 13595917 | <hkxhk> | 22.5 | 45 | 22.5 | 5 | 54 | 30 | 0.000117 | 0.123552 |
| SNP_7588 | solcap_snp_c2_56251 | chr04 | 13600249 | <hkxhk> | 22.5 | 45 | 22.5 | 27 | 36 | 25 | 0.22313 | 1 |
| SNP_6218 | solcap_snp_c2_47240 | chr04 | 29703240 | <hkxhk> | 22.5 | 45 | 22.5 | 30 | 53 | 7 | 0.000675 | 0.7128 |
| SNP_2035 | solcap_snp_c1_9476 | chr04 | 41004762 | <hkxhk> | 22.5 | 45 | 22.5 | 28 | 53 | 7 | 0.001057 | 1 |
| SNP_4278 | solcap_snp_c2_31359 | chr04 | 41356473 | <hkxhk> | 22.5 | 45 | 22.5 | 29 | 53 | 6 | 0.000388 | 0.409728 |
| SNP_2213 | solcap_snp_c2_11101 | chr04 | 42018459 | <hkxhk> | 22.5 | 45 | 22.5 | 30 | 52 | 6 | 0.000335 | 0.35376 |
| SNP_1239 | solcap_snp_c1_3436 | chr04 | 42382881 | <hkxhk> | 22.5 | 45 | 22.5 | 6 | 52 | 30 | 0.000335 | 0.35376 |
| SNP_2129 | solcap_snp_c2_10461 | chr04 | 42546608 | <hkxhk> | 22.5 | 45 | 22.5 | 6 | 53 | 29 | 0.000388 | 0.409728 |
| SNP_1472 | solcap_snp_c1_5209 | chr04 | 43831617 | <hkxhk> | 22.5 | 45 | 22.5 | 28 | 52 | 7 | 0.001194 | 1 |
| SNP_1227 | solcap_snp_c1_3319 | chr04 | 45639501 | <hkxhk> | 22.5 | 45 | 22.5 | 27 | 50 | 9 | 0.007394 | 1 |
| SNP_7652 | solcap_snp_c2_56758 | chr04 | 46061622 | <hkxhk> | 22.5 | 45 | 22.5 | 27 | 50 | 10 | 0.013662 | 1 |
| SNP_2759 | solcap_snp_c2_16720 | chr04 | 47165148 | <hkxhk> | 22.5 | 45 | 22.5 | 28 | 40 | 20 | 0.33591 | 1 |
| SNP_2758 | solcap_snp_c2_16718 | chr04 | 47529947 | <hkxhk> | 22.5 | 45 | 22.5 | 20 | 40 | 27 | 0.429631 | 1 |
| SNP_2767 | solcap_snp_c2_16744 | chr04 | 47842400 | <hkxhk> | 22.5 | 45 | 22.5 | 20 | 40 | 27 | 0.429631 | 1 |
| SNP_2629 | solcap_snp_c2_1505 | chr04 | 50735681 | <hkxhk> | 22.5 | 45 | 22.5 | 12 | 49 | 27 | 0.043936 | 1 |
| SNP_0867 | solcap_snp_c1_15878 | chr04 | 52773526 | <hkxhk> | 22.5 | 45 | 22.5 | 28 | 50 | 12 | 0.033373 | 1 |
| SNP_1679 | solcap_snp_c1_6905 | chr04 | 54530564 | <hkxhk> | 22.5 | 45 | 22.5 | 28 | 47 | 12 | 0.03979 | 1 |
| SNP_7076 | solcap_snp_c2_52884 | chr04 | 55040433 | <hkxhk> | 22.5 | 45 | 22.5 | 29 | 48 | 11 | 0.017501 | 1 |
| SNP_0781 | solcap_snp_c1_15456 | chr04 | 55345623 | <hkxhk> | 22.5 | 45 | 22.5 | 11 | 49 | 29 | 0.016647 | 1 |
| SNP_0449 | solcap_snp_c1_12945 | chr04 | 55649329 | <hkxhk> | 22.5 | 45 | 22.5 | 27 | 49 | 10 | 0.01503 | 1 |
| SNP_5786 | solcap_snp_c2_44012 | chr04 | 55704288 | <hkxhk> | 22.5 | 45 | 22.5 | 29 | 48 | 10 | 0.009902 | 1 |
| SNP_0110 | solcap_snp_c1_10762 | chr04 | 56671491 | <hkxhk> | 22.5 | 45 | 22.5 | 11 | 47 | 30 | 0.013476 | 1 |
| SNP_4867 | solcap_snp_c2_36053 | chr04 | 56746393 | <hkxhk> | 22.5 | 45 | 22.5 | 30 | 47 | 11 | 0.013476 | 1 |
| SNP_5351 | solcap_snp_c2_39817 | chr04 | 57771723 | <hkxhk> | 22.5 | 45 | 22.5 | 10 | 46 | 31 | 0.005447 | 1 |
| SNP_0791 | solcap_snp_c1_15505 | chr04 | 58220368 | <hkxhk> | 22.5 | 45 | 22.5 | 31 | 47 | 10 | 0.005429 | 1 |
| SNP_7141 | solcap_snp_c2_53548 | chr04 | 58278506 | <hkxhk> | 22.5 | 45 | 22.5 | 32 | 45 | 10 | 0.003643 | 1 |
| SNP_0181 | solcap_snp_c1_11206 | chr04 | 59738793 | <hkxhk> | 22.5 | 45 | 22.5 | 32 | 44 | 11 | 0.006252 | 1 |
| SNP_4444 | solcap_snp_c2_32601 | chr04 | 60203937 | <hkxhk> | 22.5 | 45 | 22.5 | 11 | 46 | 31 | 0.009692 | 1 |
| SNP_4439 | solcap_snp_c2_32550 | chr04 | 60357182 | <hkxhk> | 22.5 | 45 | 22.5 | 31 | 45 | 11 | 0.009567 | 1 |
| SNP_6628 | solcap_snp_c2_49997 | chr04 | 60698121 | <hkxhk> | 22.5 | 45 | 22.5 | 12 | 47 | 29 | 0.030542 | 1 |
| SNP_5295 | solcap_snp_c2_39447 | chr04 | 61187263 | <hkxhk> | 22.5 | 45 | 22.5 | 30 | 45 | 12 | 0.022917 | 1 |
| SNP_5269 | solcap_snp_c2_39327 | chr04 | 61730141 | <hkxhk> | 22.5 | 45 | 22.5 | 30 | 45 | 11 | 0.013695 | 1 |
| SNP_1658 | solcap_snp_c1_6748 | chr04 | 62390970 | <hkxhk> | 22.5 | 45 | 22.5 | 31 | 45 | 11 | 0.009567 | 1 |
| SNP_7482 | solcap_snp_c2_55768 | chr04 | 63252618 | <hkxhk> | 22.5 | 45 | 22.5 | 31 | 44 | 12 | 0.015683 | 1 |
| SNP_3750 | solcap_snp_c2_26712 | chr04 | 63324096 | <hkxhk> | 22.5 | 45 | 22.5 | 31 | 45 | 12 | 0.016163 | 1 |
| SNP_2425 | solcap_snp_c2_12981 | chr04 | 66071179 | <hkxhk> | 22.5 | 45 | 22.5 | 34 | 38 | 15 | 0.007868 | 1 |
| SNP_2422 | solcap_snp_c2_12959 | chr04 | 66197608 | <hkxhk> | 22.5 | 45 | 22.5 | 17 | 36 | 34 | 0.009902 | 1 |
| SNP_2419 | solcap_snp_c2_12956 | chr04 | 66207384 | <hkxhk> | 22.5 | 45 | 22.5 | 18 | 36 | 33 | 0.020665 | 1 |
| SNP_2414 | solcap_snp_c2_12945 | chr04 | 66243735 | <hkxhk> | 22.5 | 45 | 22.5 | 18 | 37 | 33 | 0.025464 | 1 |
| SNP_0020 | solcap_snp_c1_10168 | chr04 | 67131059 | <hkxhk> | 22.5 | 45 | 22.5 | 33 | 33 | 22 | 0.016163 | 1 |
| SNP_4623 | solcap_snp_c2_34019 | chr04 | 67168134 | <hkxhk> | 22.5 | 45 | 22.5 | 22 | 33 | 33 | 0.016163 | 1 |
| SNP_4853 | solcap_snp_c2_35970 | chr04 | 67668127 | <hkxhk> | 22.5 | 45 | 22.5 | 32 | 34 | 23 | 0.033788 | 1 |
| SNP_0090 | solcap_snp_c1_10668 | chr04 | 67813172 | <hkxhk> | 22.5 | 45 | 22.5 | 15 | 53 | 21 | 0.131586 | 1 |
| SNP_2172 | solcap_snp_c2_10819 | chr04 | 68260083 | <hkxhk> | 22.5 | 45 | 22.5 | 22 | 37 | 30 | 0.137635 | 1 |
| SNP_2164 | solcap_snp_c2_10771 | chr04 | 68373313 | <hkxhk> | 22.5 | 45 | 22.5 | 22 | 39 | 29 | 0.260684 | 1 |
| SNP_2144 | solcap_snp_c2_10617 | chr04 | 68743269 | <hkxhk> | 22.5 | 45 | 22.5 | 21 | 39 | 29 | 0.246874 | 1 |
| SNP_6952 | solcap_snp_c2_52084 | chr05 | 1960600 | <hkxhk> | 22.5 | 45 | 22.5 | 17 | 46 | 24 | 0.493172 | 1 |
| SNP_2309 | solcap_snp_c2_11747 | chr05 | 2088023 | <hkxhk> | 22.5 | 45 | 22.5 | 25 | 45 | 17 | 0.455046 | 1 |
| SNP_1294 | solcap_snp_c1_3793 | chr05 | 3721458 | <hkxhk> | 22.5 | 45 | 22.5 | 27 | 42 | 18 | 0.374277 | 1 |
| SNP_2314 | solcap_snp_c2_11829 | chr05 | 4041383 | <hkxhk> | 22.5 | 45 | 22.5 | 27 | 42 | 19 | 0.441233 | 1 |
| CDF_29 |  | chr05 | 4538880 | <hkxhk> | 22.5 | 45 | 22.5 | 27 | 45 | 17 | 0.323287 | 1 |
| SNP_6683 | solcap_snp_c2_50302 | chr05 | 4944732 | <hkxhk> | 22.5 | 45 | 22.5 | 22 | 40 | 24 | 0.774286 | 1 |
| SNP_6249 | solcap_snp_c2_47611 | chr05 | 6094634 | <hkxhk> | 22.5 | 45 | 22.5 | 25 | 42 | 20 | 0.712425 | 1 |
| SNP_5724 | solcap_snp_c2_43523 | chr05 | 9302796 | <hkxhk> | 22.5 | 45 | 22.5 | 23 | 45 | 18 | 0.681321 | 1 |
| SNP_5721 | solcap_snp_c2_43516 | chr05 | 9312213 | <hkxhk> | 22.5 | 45 | 22.5 | 23 | 40 | 27 | 0.480305 | 1 |
| SNP_2088 | solcap_snp_c1_9825 | chr05 | 10374087 | <hkxhk> | 22.5 | 45 | 22.5 | 24 | 49 | 16 | 0.309079 | 1 |
| SNP_4477 | solcap_snp_c2_32854 | chr05 | 10484269 | <hkxhk> | 22.5 | 45 | 22.5 | 24 | 49 | 14 | 0.158052 | 1 |
| SNP_0860 | solcap_snp_c1_15864 | chr05 | 11699190 | <hkxhk> | 22.5 | 45 | 22.5 | 25 | 48 | 15 | 0.22313 | 1 |
| SNP_7117 | solcap_snp_c2_53298 | chr05 | 13585000 | <hkxhk> | 22.5 | 45 | 22.5 | 26 | 46 | 15 | 0.215567 | 1 |
| SNP_6197 | solcap_snp_c2_47087 | chr05 | 13872519 | <hkxhk> | 22.5 | 45 | 22.5 | 28 | 45 | 15 | 0.143247 | 1 |
| SNP_6834 | solcap_snp_c2_5145 | chr05 | 46860445 | <hkxhk> | 22.5 | 45 | 22.5 | 26 | 49 | 15 | 0.182683 | 1 |
| SNP_7283 | solcap_snp_c2_54371 | chr05 | 52038347 | <hkxhk> | 22.5 | 45 | 22.5 | 21 | 41 | 26 | 0.613462 | 1 |
| SNP_5622 | solcap_snp_c2_42542 | chr05 | 52146668 | <hkxhk> | 22.5 | 45 | 22.5 | 27 | 40 | 21 | 0.461752 | 1 |
| SNP_8158 | solcap_snp_c2_8278 | chr05 | 53099332 | <hkxhk> | 22.5 | 45 | 22.5 | 21 | 40 | 27 | 0.461752 | 1 |
| SNP_8182 | solcap_snp_c2_8521 | chr05 | 53792626 | <hkxhk> | 22.5 | 45 | 22.5 | 28 | 40 | 19 | 0.297409 | 1 |
| SNP_8183 | solcap_snp_c2_8522 | chr05 | 53792853 | <hkxhk> | 22.5 | 45 | 22.5 | 19 | 40 | 28 | 0.297409 | 1 |
| SNP_0304 | solcap_snp_c1_1195 | chr05 | 54612535 | <hkxhk> | 22.5 | 45 | 22.5 | 18 | 44 | 26 | 0.483225 | 1 |
| SNP_0142 | solcap_snp_c1_1094 | chr05 | 54941090 | <hkxhk> | 22.5 | 45 | 22.5 | 26 | 46 | 15 | 0.215567 | 1 |
| SNP_0408 | solcap_snp_c1_1249 | chr05 | 55144745 | <hkxhk> | 22.5 | 45 | 22.5 | 25 | 47 | 15 | 0.239061 | 1 |
| SNP_4330 | solcap_snp_c2_3167 | chr06 | 4677846 | <hkxhk> | 22.5 | 45 | 22.5 | 28 | 40 | 19 | 0.297409 | 1 |
| SNP_2012 | solcap_snp_c1_925 | chr06 | 5022061 | <hkxhk> | 22.5 | 45 | 22.5 | 26 | 41 | 20 | 0.572655 | 1 |
| SNP_4950 | solcap_snp_c2_36710 | chr06 | 5921555 | <hkxhk> | 22.5 | 45 | 22.5 | 26 | 49 | 14 | 0.125803 | 1 |
| SNP_6180 | solcap_snp_c2_46994 | chr06 | 6385831 | <hkxhk> | 22.5 | 45 | 22.5 | 19 | 39 | 28 | 0.268755 | 1 |
| SNP_4613 | solcap_snp_c2_33933 | chr06 | 7398661 | <hkxhk> | 22.5 | 45 | 22.5 | 29 | 39 | 19 | 0.198902 | 1 |
| SNP_7058 | solcap_snp_c2_52798 | chr06 | 9363932 | <hkxhk> | 22.5 | 45 | 22.5 | 21 | 36 | 29 | 0.152023 | 1 |
| SNP_2052 | solcap_snp_c1_9601 | chr06 | 18783593 | <hkxhk> | 22.5 | 45 | 22.5 | 28 | 41 | 18 | 0.274417 | 1 |
| SNP_2227 | solcap_snp_c2_11287 | chr06 | 30919065 | <hkxhk> | 22.5 | 45 | 22.5 | 25 | 46 | 17 | 0.441233 | 1 |
| SNP_4487 | solcap_snp_c2_32938 | chr06 | 32504806 | <hkxhk> | 22.5 | 45 | 22.5 | 25 | 45 | 18 | 0.560152 | 1 |
| SNP_0389 | solcap_snp_c1_12409 | chr06 | 37405899 | <hkxhk> | 22.5 | 45 | 22.5 | 26 | 45 | 15 | 0.22313 | 1 |
| SNP_5391 | solcap_snp_c2_40242 | chr06 | 40713040 | <hkxhk> | 22.5 | 45 | 22.5 | 25 | 41 | 22 | 0.735784 | 1 |
| SNP_5389 | solcap_snp_c2_40236 | chr06 | 41094588 | <hkxhk> | 22.5 | 45 | 22.5 | 26 | 49 | 13 | 0.083023 | 1 |
| SNP_0971 | solcap_snp_c1_16470 | chr06 | 43205299 | <hkxhk> | 22.5 | 45 | 22.5 | 22 | 47 | 19 | 0.735784 | 1 |
| SNP_7053 | solcap_snp_c2_52763 | chr06 | 44032149 | <hkxhk> | 22.5 | 45 | 22.5 | 28 | 44 | 17 | 0.255337 | 1 |
| SNP_6266 | solcap_snp_c2_47782 | chr06 | 44774037 | <hkxhk> | 22.5 | 45 | 22.5 | 19 | 45 | 24 | 0.735784 | 1 |
| SNP_4262 | solcap_snp_c2_31156 | chr06 | 49274612 | <hkxhk> | 22.5 | 45 | 22.5 | 18 | 45 | 25 | 0.560152 | 1 |
| SNP_4844 | solcap_snp_c2_35902 | chr06 | 49472616 | <hkxhk> | 22.5 | 45 | 22.5 | 18 | 44 | 26 | 0.483225 | 1 |
| SNP_0472 | solcap_snp_c1_13155 | chr06 | 51777264 | <hkxhk> | 22.5 | 45 | 22.5 | 27 | 43 | 18 | 0.389386 | 1 |
| SNP_8209 | solcap_snp_c2_8832 | chr06 | 52066513 | <hkxhk> | 22.5 | 45 | 22.5 | 27 | 42 | 17 | 0.305426 | 1 |
| SNP_1173 | solcap_snp_c1_3000 | chr06 | 52077582 | <hkxhk> | 22.5 | 45 | 22.5 | 18 | 44 | 26 | 0.483225 | 1 |
| SNP_8200 | solcap_snp_c2_8787 | chr06 | 52357693 | <hkxhk> | 22.5 | 45 | 22.5 | 28 | 43 | 18 | 0.309079 | 1 |
| SNP_3337 | solcap_snp_c2_22301 | chr06 | 53614790 | <hkxhk> | 22.5 | 45 | 22.5 | 28 | 43 | 17 | 0.247158 | 1 |
| SNP_1695 | solcap_snp_c1_7040 | chr06 | 53910521 | <hkxhk> | 22.5 | 45 | 22.5 | 27 | 44 | 17 | 0.320984 | 1 |
| SNP_3531 | solcap_snp_c2_24082 | chr06 | 54639614 | <hkxhk> | 22.5 | 45 | 22.5 | 22 | 39 | 26 | 0.522345 | 1 |
| SNP_4062 | solcap_snp_c2_29216 | chr06 | 54898776 | <hkxhk> | 22.5 | 45 | 22.5 | 26 | 40 | 21 | 0.56611 | 1 |
| SNP_4059 | solcap_snp_c2_29187 | chr06 | 55090420 | <hkxhk> | 22.5 | 45 | 22.5 | 21 | 41 | 26 | 0.613462 | 1 |
| SNP_0173 | solcap_snp_c1_11137 | chr06 | 55716069 | <hkxhk> | 22.5 | 45 | 22.5 | 25 | 44 | 19 | 0.664253 | 1 |
| SNP_3943 | solcap_snp_c2_28107 | chr06 | 56256006 | <hkxhk> | 22.5 | 45 | 22.5 | 20 | 41 | 28 | 0.369951 | 1 |
| SNP_3703 | solcap_snp_c2_26166 | chr07 | 2573336 | <hkxhk> | 22.5 | 45 | 22.5 | 21 | 41 | 27 | 0.50673 | 1 |
| SNP_4977 | solcap_snp_c2_36835 | chr07 | 3332505 | <hkxhk> | 22.5 | 45 | 22.5 | 21 | 42 | 27 | 0.548811 | 1 |
| SNP_7106 | solcap_snp_c2_53198 | chr07 | 3537012 | <hkxhk> | 22.5 | 45 | 22.5 | 21 | 41 | 27 | 0.50673 | 1 |
| SNP_0927 | solcap_snp_c1_16223 | chr07 | 3693740 | <hkxhk> | 22.5 | 45 | 22.5 | 21 | 40 | 28 | 0.365818 | 1 |
| SNP_6089 | solcap_snp_c2_46398 | chr07 | 3992732 | <hkxhk> | 22.5 | 45 | 22.5 | 20 | 41 | 27 | 0.467029 | 1 |
| SNP_5729 | solcap_snp_c2_43574 | chr07 | 4196655 | <hkxhk> | 22.5 | 45 | 22.5 | 20 | 42 | 26 | 0.60653 | 1 |
| SNP_0301 | solcap_snp_c1_11935 | chr07 | 6096273 | <hkxhk> | 22.5 | 45 | 22.5 | 26 | 42 | 19 | 0.540671 | 1 |
| SNP_0302 | solcap_snp_c1_11937 | chr07 | 6195941 | <hkxhk> | 22.5 | 45 | 22.5 | 19 | 43 | 26 | 0.560152 | 1 |
| SNP_5624 | solcap_snp_c2_42640 | chr07 | 7400659 | <hkxhk> | 22.5 | 45 | 22.5 | 17 | 45 | 27 | 0.323287 | 1 |
| SNP_6593 | solcap_snp_c2_49842 | chr07 | 9582768 | <hkxhk> | 22.5 | 45 | 22.5 | 19 | 41 | 29 | 0.246874 | 1 |
| SNP_8015 | solcap_snp_c2_6615 | chr07 | 10249577 | <hkxhk> | 22.5 | 45 | 22.5 | 20 | 40 | 29 | 0.255337 | 1 |
| SNP_4063 | solcap_snp_c2_29253 | chr07 | 23175980 | <hkxhk> | 22.5 | 45 | 22.5 | 21 | 37 | 31 | 0.091846 | 1 |
| SNP_1158 | solcap_snp_c1_2796 | chr07 | 35378286 | <hkxhk> | 22.5 | 45 | 22.5 | 22 | 35 | 31 | 0.063205 | 1 |
| SNP_1425 | solcap_snp_c1_482 | chr07 | 38869139 | <hkxhk> | 22.5 | 45 | 22.5 | 21 | 36 | 31 | 0.074951 | 1 |
| SNP_5871 | solcap_snp_c2_4480 | chr07 | 41477381 | <hkxhk> | 22.5 | 45 | 22.5 | 21 | 38 | 29 | 0.213214 | 1 |
| SNP_1735 | solcap_snp_c1_7399 | chr07 | 44303007 | <hkxhk> | 22.5 | 45 | 22.5 | 18 | 41 | 28 | 0.274417 | 1 |
| SNP_2701 | solcap_snp_c2_16025 | chr07 | 45152653 | <hkxhk> | 22.5 | 45 | 22.5 | 19 | 39 | 29 | 0.198902 | 1 |
| SNP_0506 | solcap_snp_c1_13481 | chr07 | 46526906 | <hkxhk> | 22.5 | 45 | 22.5 | 17 | 48 | 22 | 0.471011 | 1 |
| SNP_0003 | solcap_snp_c1_10000 | chr07 | 46730348 | <hkxhk> | 22.5 | 45 | 22.5 | 18 | 42 | 28 | 0.29309 | 1 |
| SNP_5970 | solcap_snp_c2_45472 | chr07 | 47199742 | <hkxhk> | 22.5 | 45 | 22.5 | 18 | 42 | 28 | 0.29309 | 1 |
| SNP_5931 | solcap_snp_c2_45180 | chr07 | 48177745 | <hkxhk> | 22.5 | 45 | 22.5 | 17 | 48 | 25 | 0.402077 | 1 |
| SNP_3103 | solcap_snp_c2_19826 | chr07 | 50146448 | <hkxhk> | 22.5 | 45 | 22.5 | 26 | 47 | 15 | 0.206068 | 1 |
| SNP_0059 | solcap_snp_c1_10461 | chr07 | 50712704 | <hkxhk> | 22.5 | 45 | 22.5 | 15 | 48 | 26 | 0.194985 | 1 |
| SNP_4753 | solcap_snp_c2_35053 | chr07 | 50790045 | <hkxhk> | 22.5 | 45 | 22.5 | 26 | 46 | 16 | 0.29309 | 1 |
| SNP_3683 | solcap_snp_c2_26003 | chr07 | 51200564 | <hkxhk> | 22.5 | 45 | 22.5 | 18 | 47 | 23 | 0.613462 | 1 |
| SNP_2095 | solcap_snp_c1_9878 | chr07 | 51991553 | <hkxhk> | 22.5 | 45 | 22.5 | 21 | 43 | 24 | 0.882496 | 1 |
| SNP_4511 | solcap_snp_c2_33038 | chr07 | 52107222 | <hkxhk> | 22.5 | 45 | 22.5 | 17 | 47 | 24 | 0.467029 | 1 |
| SNP_1940 | solcap_snp_c1_8686 | chr07 | 52469689 | <hkxhk> | 22.5 | 45 | 22.5 | 22 | 46 | 20 | 0.872525 | 1 |
| SNP_5641 | solcap_snp_c2_42763 | chr07 | 53172106 | <hkxhk> | 22.5 | 45 | 22.5 | 22 | 43 | 23 | 0.966483 | 1 |
| SNP_2352 | solcap_snp_c2_12405 | chr07 | 53517986 | <hkxhk> | 22.5 | 45 | 22.5 | 25 | 39 | 24 | 0.560152 | 1 |
| SNP_2958 | solcap_snp_c2_18570 | chr07 | 55877066 | <hkxhk> | 22.5 | 45 | 22.5 | 21 | 48 | 19 | 0.664253 | 1 |
| SNP_1957 | solcap_snp_c1_8819 | chr07 | 56679017 | <hkxhk> | 22.5 | 45 | 22.5 | 18 | 48 | 20 | 0.533707 | 1 |
| SNP_4019 | solcap_snp_c2_28849 | chr07 | 56784999 | <hkxhk> | 22.5 | 45 | 22.5 | 19 | 48 | 22 | 0.686324 | 1 |
| SNP_4026 | solcap_snp_c2_28875 | chr07 | 56864186 | <hkxhk> | 22.5 | 45 | 22.5 | 18 | 50 | 19 | 0.374277 | 1 |
| SNP_1958 | solcap_snp_c1_8829 | chr07 | 56928346 | <hkxhk> | 22.5 | 45 | 22.5 | 20 | 49 | 18 | 0.476456 | 1 |
| SNP_4529 | solcap_snp_c2_33278 | chr07 | 57510476 | <hkxhk> | 22.5 | 45 | 22.5 | 17 | 51 | 20 | 0.29644 | 1 |
| SNP_3830 | solcap_snp_c2_27452 | chr08 | 2673901 | <hkxhk> | 22.5 | 45 | 22.5 | 21 | 48 | 18 | 0.56611 | 1 |
| SNP_3833 | solcap_snp_c2_27473 | chr08 | 2786806 | <hkxhk> | 22.5 | 45 | 22.5 | 22 | 45 | 20 | 0.906919 | 1 |
| SNP_3555 | solcap_snp_c2_24403 | chr08 | 4088758 | <hkxhk> | 22.5 | 45 | 22.5 | 31 | 39 | 17 | 0.06598 | 1 |
| SNP_4628 | solcap_snp_c2_34103 | chr08 | 5156455 | <hkxhk> | 22.5 | 45 | 22.5 | 32 | 41 | 14 | 0.020903 | 1 |
| SNP_4144 | solcap_snp_c2_30053 | chr08 | 5752069 | <hkxhk> | 22.5 | 45 | 22.5 | 9 | 45 | 34 | 0.000804 | 0.849024 |
| SNP_4148 | solcap_snp_c2_30067 | chr08 | 5899089 | <hkxhk> | 22.5 | 45 | 22.5 | 36 | 45 | 9 | 0.000303 | 0.319968 |
| SNP_7070 | solcap_snp_c2_52856 | chr08 | 8095300 | <hkxhk> | 22.5 | 45 | 22.5 | 21 | 50 | 17 | 0.367879 | 1 |
| SNP_1594 | solcap_snp_c1_6130 | chr08 | 20343694 | <hkxhk> | 22.5 | 45 | 22.5 | 10 | 41 | 37 | 0.000205 | 0.21648 |
| SNP_3111 | solcap_snp_c2_19934 | chr08 | 30356352 | <hkxhk> | 22.5 | 45 | 22.5 | 10 | 42 | 36 | 0.000421 | 0.444576 |
| SNP_3275 | solcap_snp_c2_2178 | chr08 | 32200861 | <hkxhk> | 22.5 | 45 | 22.5 | 36 | 42 | 10 | 0.000421 | 0.444576 |
| SNP_4692 | solcap_snp_c2_34564 | chr08 | 33944300 | <hkxhk> | 22.5 | 45 | 22.5 | 18 | 47 | 22 | 0.62781 | 1 |
| SNP_7190 | solcap_snp_c2_53880 | chr08 | 34672021 | <hkxhk> | 22.5 | 45 | 22.5 | 11 | 41 | 36 | 0.00067 | 0.70752 |
| SNP_4165 | solcap_snp_c2_30254 | chr08 | 35824716 | <hkxhk> | 22.5 | 45 | 22.5 | 36 | 41 | 11 | 0.00067 | 0.70752 |
| SNP_4472 | solcap_snp_c2_32802 | chr08 | 38420122 | <hkxhk> | 22.5 | 45 | 22.5 | 35 | 42 | 11 | 0.001311 | 1 |
| SNP_0351 | solcap_snp_c1_12162 | chr08 | 39547409 | <hkxhk> | 22.5 | 45 | 22.5 | 19 | 48 | 20 | 0.620635 | 1 |
| SNP_5825 | solcap_snp_c2_44305 | chr08 | 42730456 | <hkxhk> | 22.5 | 45 | 22.5 | 11 | 47 | 31 | 0.009707 | 1 |
| SNP_5828 | solcap_snp_c2_44334 | chr08 | 42836681 | <hkxhk> | 22.5 | 45 | 22.5 | 22 | 42 | 24 | 0.872525 | 1 |
| SNP_0722 | solcap_snp_c1_15044 | chr08 | 42972745 | <hkxhk> | 22.5 | 45 | 22.5 | 23 | 40 | 25 | 0.664253 | 1 |
| SNP_6236 | solcap_snp_c2_47459 | chr08 | 43188506 | <hkxhk> | 22.5 | 45 | 22.5 | 10 | 50 | 29 | 0.008774 | 1 |
| SNP_4537 | solcap_snp_c2_33381 | chr08 | 43849684 | <hkxhk> | 22.5 | 45 | 22.5 | 25 | 38 | 25 | 0.441233 | 1 |
| SNP_0352 | solcap_snp_c1_12166 | chr08 | 44459320 | <hkxhk> | 22.5 | 45 | 22.5 | 25 | 37 | 25 | 0.378604 | 1 |
| SNP_5394 | solcap_snp_c2_40290 | chr08 | 49554337 | <hkxhk> | 22.5 | 45 | 22.5 | 10 | 46 | 31 | 0.005447 | 1 |
| SNP_3979 | solcap_snp_c2_28521 | chr08 | 50650322 | <hkxhk> | 22.5 | 45 | 22.5 | 32 | 44 | 11 | 0.006252 | 1 |
| SNP_3997 | solcap_snp_c2_28633 | chr08 | 51201973 | <hkxhk> | 22.5 | 45 | 22.5 | 12 | 48 | 28 | 0.037902 | 1 |
| SNP_0468 | solcap_snp_c1_13116 | chr08 | 51625129 | <hkxhk> | 22.5 | 45 | 22.5 | 13 | 49 | 27 | 0.070137 | 1 |
| SNP_4708 | solcap_snp_c2_34705 | chr08 | 52840357 | <hkxhk> | 22.5 | 45 | 22.5 | 15 | 48 | 26 | 0.194985 | 1 |
| SNP_4955 | solcap_snp_c2_36731 | chr08 | 53441680 | <hkxhk> | 22.5 | 45 | 22.5 | 18 | 44 | 27 | 0.400224 | 1 |
| SNP_4965 | solcap_snp_c2_36780 | chr08 | 53661470 | <hkxhk> | 22.5 | 45 | 22.5 | 19 | 43 | 27 | 0.463169 | 1 |
| SNP_6381 | solcap_snp_c2_48441 | chr08 | 53748757 | <hkxhk> | 22.5 | 45 | 22.5 | 20 | 42 | 27 | 0.501069 | 1 |
| SNP_3031 | solcap_snp_c2_19085 | chr08 | 54954128 | <hkxhk> | 22.5 | 45 | 22.5 | 19 | 43 | 27 | 0.463169 | 1 |
| SNP_3018 | solcap_snp_c2_19017 | chr08 | 55202444 | <hkxhk> | 22.5 | 45 | 22.5 | 19 | 44 | 26 | 0.573395 | 1 |
| SNP_3019 | solcap_snp_c2_19018 | chr08 | 55202475 | <hkxhk> | 22.5 | 45 | 22.5 | 26 | 44 | 19 | 0.573395 | 1 |
| SNP_4700 | solcap_snp_c2_34634 | chr08 | 55926220 | <hkxhk> | 22.5 | 45 | 22.5 | 21 | 41 | 25 | 0.720661 | 1 |
| SNP_3968 | solcap_snp_c2_28475 | chr08 | 59145557 | <hkxhk> | 22.5 | 45 | 22.5 | 24 | 42 | 23 | 0.859258 | 1 |
| SNP_4546 | solcap_snp_c2_3345 | chr09 | 249687 | <hkxhk> | 22.5 | 45 | 22.5 | 16 | 43 | 28 | 0.189964 | 1 |
| SNP_4517 | solcap_snp_c2_3318 | chr09 | 279397 | <hkxhk> | 22.5 | 45 | 22.5 | 16 | 44 | 27 | 0.247448 | 1 |
| SNP_0002 | solcap_snp_c1_1000 | chr09 | 526911 | <hkxhk> | 22.5 | 45 | 22.5 | 14 | 46 | 27 | 0.124157 | 1 |
| SNP_0625 | solcap_snp_c1_14393 | chr09 | 755762 | <hkxhk> | 22.5 | 45 | 22.5 | 14 | 44 | 30 | 0.054525 | 1 |
| SNP_1264 | solcap_snp_c1_3597 | chr09 | 1937916 | <hkxhk> | 22.5 | 45 | 22.5 | 15 | 44 | 28 | 0.142519 | 1 |
| SNP_2061 | solcap_snp_c1_9652 | chr09 | 3195965 | <hkxhk> | 22.5 | 45 | 22.5 | 15 | 44 | 28 | 0.142519 | 1 |
| SNP_0551 | solcap_snp_c1_1383 | chr09 | 3648430 | <hkxhk> | 22.5 | 45 | 22.5 | 16 | 44 | 28 | 0.194686 | 1 |
| SNP_5525 | solcap_snp_c2_4165 | chr09 | 3915501 | <hkxhk> | 22.5 | 45 | 22.5 | 28 | 43 | 16 | 0.189964 | 1 |
| SNP_2450 | solcap_snp_c2_13293 | chr09 | 5896700 | <hkxhk> | 22.5 | 45 | 22.5 | 26 | 47 | 15 | 0.206068 | 1 |
| SNP_1354 | solcap_snp_c1_4248 | chr09 | 6218953 | <hkxhk> | 22.5 | 45 | 22.5 | 17 | 43 | 28 | 0.247158 | 1 |
| SNP_1349 | solcap_snp_c1_4228 | chr09 | 6515616 | <hkxhk> | 22.5 | 45 | 22.5 | 28 | 43 | 17 | 0.247158 | 1 |
| SNP_0682 | solcap_snp_c1_14783 | chr09 | 7354603 | <hkxhk> | 22.5 | 45 | 22.5 | 16 | 46 | 27 | 0.244115 | 1 |
| SNP_0546 | solcap_snp_c1_13785 | chr09 | 8432571 | <hkxhk> | 22.5 | 45 | 22.5 | 18 | 44 | 26 | 0.483225 | 1 |
| SNP_0735 | solcap_snp_c1_1512 | chr09 | 8783890 | <hkxhk> | 22.5 | 45 | 22.5 | 26 | 45 | 16 | 0.300848 | 1 |
| SNP_7335 | solcap_snp_c2_54613 | chr09 | 8898293 | <hkxhk> | 22.5 | 45 | 22.5 | 16 | 45 | 26 | 0.300848 | 1 |
| SNP_0668 | solcap_snp_c1_14676 | chr09 | 10232571 | <hkxhk> | 22.5 | 45 | 22.5 | 17 | 43 | 27 | 0.315003 | 1 |
| SNP_7899 | solcap_snp_c2_58234 | chr09 | 11085152 | <hkxhk> | 22.5 | 45 | 22.5 | 28 | 45 | 15 | 0.143247 | 1 |
| SNP_7084 | solcap_snp_c2_52898 | chr09 | 11180844 | <hkxhk> | 22.5 | 45 | 22.5 | 15 | 45 | 28 | 0.143247 | 1 |
| SNP_6041 | solcap_snp_c2_46028 | chr09 | 12485179 | <hkxhk> | 22.5 | 45 | 22.5 | 25 | 48 | 16 | 0.305625 | 1 |
| SNP_4585 | solcap_snp_c2_33626 | chr09 | 14531441 | <hkxhk> | 22.5 | 45 | 22.5 | 25 | 46 | 17 | 0.441233 | 1 |
| SNP_2038 | solcap_snp_c1_9490 | chr09 | 15271506 | <hkxhk> | 22.5 | 45 | 22.5 | 26 | 46 | 16 | 0.29309 | 1 |
| SNP_4293 | solcap_snp_c2_31383 | chr09 | 15463547 | <hkxhk> | 22.5 | 45 | 22.5 | 26 | 44 | 17 | 0.391887 | 1 |
| SNP_7374 | solcap_snp_c2_549 | chr09 | 16260436 | <hkxhk> | 22.5 | 45 | 22.5 | 18 | 46 | 25 | 0.548195 | 1 |
| SNP_1078 | solcap_snp_c1_216 | chr09 | 36013994 | <hkxhk> | 22.5 | 45 | 22.5 | 25 | 47 | 17 | 0.423352 | 1 |
| SNP_1074 | solcap_snp_c1_212 | chr09 | 36830823 | <hkxhk> | 22.5 | 45 | 22.5 | 17 | 64 | 9 | 0.000161 | 0.170016 |
| SNP_2715 | solcap_snp_c2_16275 | chr09 | 48595888 | <hkxhk> | 22.5 | 45 | 22.5 | 24 | 43 | 21 | 0.882496 | 1 |
| SNP_3160 | solcap_snp_c2_20479 | chr09 | 51133803 | <hkxhk> | 22.5 | 45 | 22.5 | 24 | 43 | 22 | 0.908913 | 1 |
| SNP_2384 | solcap_snp_c2_12761 | chr09 | 52547193 | <hkxhk> | 22.5 | 45 | 22.5 | 22 | 42 | 23 | 0.938738 | 1 |
| SNP_1326 | solcap_snp_c1_4078 | chr09 | 52667666 | <hkxhk> | 22.5 | 45 | 22.5 | 21 | 45 | 23 | 0.950695 | 1 |
| SNP_5873 | solcap_snp_c2_44815 | chr09 | 53270483 | <hkxhk> | 22.5 | 45 | 22.5 | 20 | 50 | 18 | 0.421626 | 1 |
| SNP_2538 | solcap_snp_c2_14636 | chr09 | 56854476 | <hkxhk> | 22.5 | 45 | 22.5 | 24 | 45 | 20 | 0.830777 | 1 |
| SNP_5550 | solcap_snp_c2_4197 | chr09 | 58311901 | <hkxhk> | 22.5 | 45 | 22.5 | 23 | 45 | 21 | 0.950695 | 1 |
| SNP_5451 | solcap_snp_c2_40867 | chr09 | 58855271 | <hkxhk> | 22.5 | 45 | 22.5 | 21 | 46 | 21 | 0.9131 | 1 |
| SNP_6822 | solcap_snp_c2_51346 | chr09 | 59026771 | <hkxhk> | 22.5 | 45 | 22.5 | 23 | 45 | 21 | 0.950695 | 1 |
| SNP_1681 | solcap_snp_c1_6936 | chr09 | 60515757 | <hkxhk> | 22.5 | 45 | 22.5 | 16 | 49 | 22 | 0.329824 | 1 |
| SNP_3298 | solcap_snp_c2_21999 | chr09 | 60718463 | <hkxhk> | 22.5 | 45 | 22.5 | 14 | 49 | 26 | 0.125803 | 1 |
| SNP_3302 | solcap_snp_c2_22033 | chr09 | 60916970 | <hkxhk> | 22.5 | 45 | 22.5 | 15 | 50 | 23 | 0.213214 | 1 |
| SNP_3306 | solcap_snp_c2_22054 | chr09 | 61033762 | <hkxhk> | 22.5 | 45 | 22.5 | 13 | 51 | 25 | 0.076733 | 1 |
| SNP_3307 | solcap_snp_c2_22067 | chr09 | 61110733 | <hkxhk> | 22.5 | 45 | 22.5 | 23 | 50 | 14 | 0.149225 | 1 |
| SNP_3310 | solcap_snp_c2_22072 | chr09 | 61134494 | <hkxhk> | 22.5 | 45 | 22.5 | 14 | 50 | 25 | 0.130116 | 1 |
| SNP_6147 | solcap_snp_c2_46777 | chr09 | 61497547 | <hkxhk> | 22.5 | 45 | 22.5 | 30 | 40 | 19 | 0.162901 | 1 |
| SNP_4138 | solcap_snp_c2_29945 | chr09 | 62330939 | <hkxhk> | 22.5 | 45 | 22.5 | 23 | 51 | 14 | 0.130799 | 1 |
| SNP_2500 | solcap_snp_c2_13969 | chr09 | 62842641 | <hkxhk> | 22.5 | 45 | 22.5 | 30 | 39 | 20 | 0.164742 | 1 |
| SNP_3183 | solcap_snp_c2_20698 | chr09 | 63372364 | <hkxhk> | 22.5 | 45 | 22.5 | 14 | 50 | 24 | 0.141628 | 1 |
| SNP_5364 | solcap_snp_c2_40032 | chr09 | 63515478 | <hkxhk> | 22.5 | 45 | 22.5 | 14 | 50 | 23 | 0.149225 | 1 |
| SNP_5372 | solcap_snp_c2_40085 | chr09 | 63676068 | <hkxhk> | 22.5 | 45 | 22.5 | 29 | 40 | 20 | 0.255337 | 1 |
| SNP_1996 | solcap_snp_c1_914 | chr09 | 64411443 | <hkxhk> | 22.5 | 45 | 22.5 | 22 | 50 | 15 | 0.215567 | 1 |
| SNP_0233 | solcap_snp_c1_11517 | chr09 | 67070635 | <hkxhk> | 22.5 | 45 | 22.5 | 18 | 40 | 29 | 0.187793 | 1 |
| SNP_2107 | solcap_snp_c2_1001 | chr10 | 1789546 | <hkxhk> | 22.5 | 45 | 22.5 | 18 | 47 | 22 | 0.62781 | 1 |
| SNP_2181 | solcap_snp_c2_1092 | chr10 | 2229270 | <hkxhk> | 22.5 | 45 | 22.5 | 19 | 43 | 26 | 0.560152 | 1 |
| SNP_0912 | solcap_snp_c1_16148 | chr10 | 4566768 | <hkxhk> | 22.5 | 45 | 22.5 | 31 | 40 | 16 | 0.056822 | 1 |
| SNP_0281 | solcap_snp_c1_11801 | chr10 | 5685940 | <hkxhk> | 22.5 | 45 | 22.5 | 18 | 38 | 31 | 0.071509 | 1 |
| SNP_3658 | solcap_snp_c2_25510 | chr10 | 49532339 | <hkxhk> | 22.5 | 45 | 22.5 | 6 | 46 | 35 | 5.49E-05 | 1 |
| SNP_7094 | solcap_snp_c2_53064 | chr10 | 50731859 | <hkxhk> | 22.5 | 45 | 22.5 | 35 | 47 | 5 | 2.43E-05 | 1 |
| SNP_2455 | solcap_snp_c2_13350 | chr11 | 723105 | <hkxhk> | 22.5 | 45 | 22.5 | 24 | 42 | 21 | 0.856267 | 1 |
| SNP_1364 | solcap_snp_c1_4347 | chr11 | 1462181 | <hkxhk> | 22.5 | 45 | 22.5 | 22 | 43 | 22 | 0.994269 | 1 |
| SNP_5027 | solcap_snp_c2_37201 | chr11 | 2565871 | <hkxhk> | 22.5 | 45 | 22.5 | 23 | 43 | 22 | 0.966483 | 1 |
| SNP_4592 | solcap_snp_c2_33678 | chr11 | 3211810 | <hkxhk> | 22.5 | 45 | 22.5 | 22 | 44 | 22 | 1 | 1 |
| SNP_1079 | solcap_snp_c1_2162 | chr11 | 3322959 | <hkxhk> | 22.5 | 45 | 22.5 | 22 | 44 | 22 | 1 | 1 |
| SNP_1089 | solcap_snp_c1_2280 | chr11 | 4601740 | <hkxhk> | 22.5 | 45 | 22.5 | 24 | 42 | 22 | 0.872525 | 1 |
| SNP_4028 | solcap_snp_c2_2896 | chr11 | 8427832 | <hkxhk> | 22.5 | 45 | 22.5 | 23 | 41 | 24 | 0.805808 | 1 |
| SNP_7637 | solcap_snp_c2_56630 | chr11 | 11020286 | <hkxhk> | 22.5 | 45 | 22.5 | 23 | 45 | 19 | 0.790071 | 1 |
| SNP_4051 | solcap_snp_c2_29113 | chr11 | 11881477 | <hkxhk> | 22.5 | 45 | 22.5 | 19 | 45 | 23 | 0.790071 | 1 |
| SNP_3061 | solcap_snp_c2_19461 | chr11 | 15130283 | <hkxhk> | 22.5 | 45 | 22.5 | 20 | 44 | 25 | 0.750874 | 1 |
| SNP_4113 | solcap_snp_c2_2968 | chr11 | 30014937 | <hkxhk> | 22.5 | 45 | 22.5 | 25 | 38 | 23 | 0.533707 | 1 |
| SNP_6778 | solcap_snp_c2_50980 | chr11 | 31864206 | <hkxhk> | 22.5 | 45 | 22.5 | 23 | 40 | 23 | 0.811149 | 1 |
| SNP_0669 | solcap_snp_c1_1468 | chr11 | 33199798 | <hkxhk> | 22.5 | 45 | 22.5 | 25 | 47 | 14 | 0.168795 | 1 |
| SNP_5632 | solcap_snp_c2_4275 | chr11 | 33453799 | <hkxhk> | 22.5 | 45 | 22.5 | 25 | 50 | 13 | 0.085902 | 1 |
| SNP_4915 | solcap_snp_c2_36587 | chr11 | 34090279 | <hkxhk> | 22.5 | 45 | 22.5 | 26 | 48 | 13 | 0.08999 | 1 |
| SNP_0337 | solcap_snp_c1_12078 | chr11 | 35462389 | <hkxhk> | 22.5 | 45 | 22.5 | 27 | 47 | 13 | 0.079302 | 1 |
| SNP_4303 | solcap_snp_c2_31444 | chr11 | 36343765 | <hkxhk> | 22.5 | 45 | 22.5 | 26 | 50 | 12 | 0.047574 | 1 |
| SNP_1366 | solcap_snp_c1_4371 | chr11 | 37893716 | <hkxhk> | 22.5 | 45 | 22.5 | 26 | 49 | 12 | 0.05243 | 1 |
| SNP_2470 | solcap_snp_c2_13627 | chr11 | 38340219 | <hkxhk> | 22.5 | 45 | 22.5 | 26 | 50 | 11 | 0.02851 | 1 |
| SNP_5939 | solcap_snp_c2_45206 | chr11 | 38846070 | <hkxhk> | 22.5 | 45 | 22.5 | 25 | 51 | 12 | 0.048118 | 1 |
| SNP_6855 | solcap_snp_c2_51546 | chr11 | 39067742 | <hkxhk> | 22.5 | 45 | 22.5 | 25 | 49 | 12 | 0.060668 | 1 |
| SNP_6581 | solcap_snp_c2_49808 | chr11 | 39730037 | <hkxhk> | 22.5 | 45 | 22.5 | 25 | 48 | 13 | 0.104788 | 1 |
| SNP_2597 | solcap_snp_c2_14946 | chr11 | 39858654 | <hkxhk> | 22.5 | 45 | 22.5 | 13 | 48 | 26 | 0.08999 | 1 |
| SNP_3324 | solcap_snp_c2_22189 | chr11 | 42274331 | <hkxhk> | 22.5 | 45 | 22.5 | 15 | 50 | 23 | 0.213214 | 1 |
| SNP_2644 | solcap_snp_c2_15329 | chr11 | 43017275 | <hkxhk> | 22.5 | 45 | 22.5 | 25 | 38 | 25 | 0.441233 | 1 |
| SNP_4170 | solcap_snp_c2_30297 | chr11 | 44107400 | <hkxhk> | 22.5 | 45 | 22.5 | 20 | 51 | 16 | 0.228319 | 1 |
| SNP_4310 | solcap_snp_c2_31487 | chr11 | 45324790 | <hkxhk> | 22.5 | 45 | 22.5 | 24 | 39 | 25 | 0.560152 | 1 |
| SNP_5756 | solcap_snp_c2_43860 | chr11 | 45821838 | <hkxhk> | 22.5 | 45 | 22.5 | 23 | 41 | 24 | 0.805808 | 1 |
| SNP_0036 | solcap_snp_c1_10256 | chr11 | 46391373 | <hkxhk> | 22.5 | 45 | 22.5 | 22 | 43 | 23 | 0.966483 | 1 |
| SNP_7341 | solcap_snp_c2_5463 | chr12 | 626165 | <hkxhk> | 22.5 | 45 | 22.5 | 21 | 51 | 16 | 0.247158 | 1 |
| SNP_7558 | solcap_snp_c2_5595 | chr12 | 1132071 | <hkxhk> | 22.5 | 45 | 22.5 | 15 | 49 | 25 | 0.206253 | 1 |
| SNP_0536 | solcap_snp_c1_13698 | chr12 | 2428946 | <hkxhk> | 22.5 | 45 | 22.5 | 27 | 48 | 14 | 0.113703 | 1 |
| SNP_1096 | solcap_snp_c1_2331 | chr12 | 4424263 | <hkxhk> | 22.5 | 45 | 22.5 | 30 | 45 | 14 | 0.056021 | 1 |
| SNP_7755 | solcap_snp_c2_57400 | chr12 | 6109094 | <hkxhk> | 22.5 | 45 | 22.5 | 31 | 41 | 18 | 0.10717 | 1 |
| SNP_0829 | solcap_snp_c1_15695 | chr12 | 51388931 | <hkxhk> | 22.5 | 45 | 22.5 | 17 | 41 | 30 | 0.119432 | 1 |
| SNP_4717 | solcap_snp_c2_34776 | chr12 | 51982134 | <hkxhk> | 22.5 | 45 | 22.5 | 17 | 40 | 31 | 0.074951 | 1 |
| SNP_3600 | solcap_snp_c2_24654 | chr12 | 53396676 | <hkxhk> | 22.5 | 45 | 22.5 | 18 | 39 | 31 | 0.083023 | 1 |
| SNP_6078 | solcap_snp_c2_46299 | chr12 | 54478428 | <hkxhk> | 22.5 | 45 | 22.5 | 18 | 41 | 29 | 0.206068 | 1 |
| SNP_5747 | solcap_snp_c2_43774 | chr12 | 56337816 | <hkxhk> | 22.5 | 45 | 22.5 | 23 | 34 | 31 | 0.049787 | 1 |
| SNP_1784 | solcap_snp_c1_7754 | chr12 | 59290871 | <hkxhk> | 22.5 | 45 | 22.5 | 17 | 52 | 19 | 0.22313 | 1 |
| SNP_3569 | solcap_snp_c2_24535 | chr12 | 59370346 | <hkxhk> | 22.5 | 45 | 22.5 | 23 | 37 | 26 | 0.389901 | 1 |

**Supplementary Table 6**. StCDF1 KASP^TM^ genotyping markers. The sequence around the primers used for the KASP assay is listed below. The nucleotides within the square bracket show the sequence variation between the two alleles with the FAM allele on the left and HEX allele on the right of the “/”. Nucleotides indicated bold and ending with a green nucleotide are primer X. Primer Y is the same as primer X only the red nucleotide is substituted for the green.

| **Assays ID** | **FAM Allele** | **HEX Allele** | **Sequence** | **Primer Seq Allele X** | **Primer Seq Allele Y** | **Primer Seq common** |
| --- | --- | --- | --- | --- | --- | --- |
| snpST00091 | CACTAGG | none | …TTGCAGAATAGAGAAGGCGAGAGATGTGTACTGATTCCAAAGACATTAAGGATTCATGATCCAAATGAAGCGGCTAAAAGCTCTATATGGTCAACACTAGG[**C**ACTAGG/]**T**ATCAGGAATGAGAAGATTGATTCGGCTCGTGGTACAATGCTCTTCAGTGCCTTCAATCCAAAAGCTGATCATAGAAATCGCGAACATGACACTTCTTTTG… | AAGCTCTATATGGTCAACACTAGGC | AAAAGCTCTATATGGTCAACACTAGGT | CCACGAGCCGAATCAATCTTCTCAT |
| snpST00092 | *865 bp insertion | none | …TTGCAGAATAGAGAAGGCGAGAGATGTGTACTGATTCCAAAGACATTAAGGATTCATGATCCAAATGAAGCGGCTAAAAGCTCTATATGGTCAACACTAGGTA[**A***/]**T**CAGGAATGAGAAGATTGATTCGGCTCGTGGTACAATGCTCTTCAGTGCCTTCAATCCAAAAGCTGATCATAGAAATCGCGAACATGACACTTCTTTTG… | AAGCTCTATATGGTCAACACTAGGTAA | AAGCTCTATATGGTCAACACTAGGTAT | CCACGAGCCGAATCAATCTTCTCAT |
| snpST00110 | G | A | …CATTCTATCCAGCAACACCGTACTGGGGCTGCACCGTAGCAAACCCTTGGAACGTACCTTGGCTTTCTTCTGATCAATC[G/A]TCAGTCCAGAACAACAGTCCTACTTCACCAACATTAGGAAAACATTCTCGGGATGAAAGC… | TAGGACTGTTGTTCTGGACTGAC | AGTAGGACTGTTGTTCTGGACTGAT | AACGTACCTTGGCTTTCTTCTGATCAAT |

*865 bp insertion: AAGGCTGGGCACCGGACCGGAATGGGACCACCGGACCGGAACGAACCGGAACCGGACCGGAACGGGACGGTTTGACCGGGTTGTTGACCGGTACCGGGATGAACCGGACCGGAACTACCGGGATGGAGGCTCGGTTCCGTCCCGTCCCACTATATACCGGGATAGAACCGGGACGGACCGGAATGGACCGGAACGGAACGGGATGGGATAAACGGGACGGCACATATAGCTATTTAAAAAAAAAATTATTTTTTTTGAGTTATTTTGAATTACGAAAATACGAATGTTTTTTTTATTTTTCTAAGTTATATTAGTTTATAGTATTTAAGTTTTAAAATTTATAATAATTTTACTTAAGTTTATTTTAAAGATATTTTTTTTAATTTTTACGTATATAAGTTTGTAAGTTAAGTTTAATAAAGTTTTTTTTTTAGTTTTTGAGCTTATGTAGTTATGTTATAAGTTTTAAAGATTTGAATCTTTTGAAGTTTATAAGTTATTAAGTTATACTTATAACTTGTAAGTTAAATAACTTAAGTTTGTAATTTTAAAAAAATTAAATAAACAAAAAAGAAATGCTAATAAAAGTAAATAATGACAAAAATATTATTTTTTATTTTAATTAAAAATGATTTACCGGGACCGGACCGGTACCGGACCGGACCGGAACGGAACCGGAATGAACCGGGATGAACCGGTACCGGTATACCGGTACCAAATCATGGTACCGTTCCGTTCCGTGTACCGGTTCAATGTATCCCATCCCGTCCCGAATACTACCGGACCGGAACGAACCGGAATGGTACCGGAACGGACCGGAATGGTACCGGTACAACCCGTTCCGGTGCCCAGCCTTAACACTAGG

**Supplementary Table 7**. StCDF1 KASP assay genotyping calls for diploid clones. 15143-xxT clones were progeny from parents 12120-03 X 07506-01.

| **clone ID^a^** | **HRM *StCDF1*^b^** | **snpST00110^c^** | **snpST00091^d^** | **snpST00092^e^** |
| --- | --- | --- | --- | --- |
| 15143-20T | StCDF1.1: StCDF1.1 | G:G | present | present |
| 15143-25T | StCDF1.3: StCDF1.3 | A:A |  |  |
| 15143-29T | StCDF1.3: StCDF1.3 | A:A |  |  |
| 15143-33T | StCDF1.1: StCDF1.1 | G:G | present | present |
| 15143-34T | StCDF1.3: StCDF1.1 | A:G | present |  |
| 15143-40T | StCDF1.1: StCDF1.1 | G:G | present | present |
| 15143-56T | StCDF1.3: StCDF1.1 | A:G | present |  |
| 15143-58T | StCDF1.1: StCDF1.1 | G:G | present | present |
| 15143-59T | StCDF1.3: StCDF1.3 | A:A |  |  |
| 15143-60T | StCDF1.3: StCDF1.1 | A:G | present |  |
| 15143-79T | StCDF1.3: StCDF1.1 | A:G | present |  |
| 07506-01 | StCDF1.3: StCDF1.1 | A:G | present |  |
| 12120-03 | StCDF1.3: StCDF1.1 | A:G | present |  |
| 12625-02 | Not done | A:A | present | present |
| 10908-06 | Not done | A:A | present |  |
| 11379-03 | Not done | A:A | present | present |
| 08675-21 | Not done | A:G | present |  |
| H412-1 | Not done | A:A | present | present |
| W5281.2 | Not done | A:G | present |  |
| DW84-1457 | Not done | A:A |  |  |

**^a^** The 15143-xxT clones are progeny from a cross of 12120-03 x 07506-01. Previous sequencing information indicated only StCDF1.1 and StCDF1.3 are present in these lines.

**^b^** Previous sequencing of the 07506-01 and 12120-03 StCDF1 alleles demonstrated that A allele was associated with StCDF1.3 and the G allele with StCDF1.1, which can be detected using a high resolution melting (HRM) assay (Tai et al. 2018). However, some other lines, such as DM-1, show that StCDF1.1 alleles also have an A in this position. Therefore, SNP for detection of StCDF1 using the SNP will be for lines where there is sequence information for the StCDF1 alleles of the parents that supports its use.

**^c^** snpST00110 KASP genotyping assay calls are for the same SNP as for the HRM StCDF1 assay.

**^d^** The KASP genotyping assay detects the presence of the snpST00091 primer Y and common primer PCR products amplified from StCDF1.1 or StCDF1.4 alleles. The StCDF1.2 allele is not found in the clones examined, therefore the snpST00091 primer X PCR product will not be formed. In this case, the genotyping calling is present/absent of the StCDF1.1 or StCDF1.4 allele.

**^e^**  The snpST00092 primer X and common primer PCR products amplified from the StCDF1.3 allele produce a product that forms a stable secondary structure (Supplementary Fig. 6). This structure interferes with PCR amplification in the KASP genotyping assay. The snpST00092 primer Y and common primer PCR product amplified from the StCDF1.1 or StCDF1.4 allele do not form a stable secondary structure (Supplementary Table 6), however, observations from the 15143-xxT clones indicate that primer Y PCR products are also not formed in the presence of the StCDF1.3 allele. The snpST00092 primer Y PCR products were only observed in the absence of StCDF1.3. Therefore, presence calls are for StCDF1.1 and StCDF1.4 alleles only in absence of StCDF1.3.
